# Overview of the Alaskan Layered Pollution and Chemical Analysis (ALPACA) Field Experiment

**DOI:** 10.1021/acsestair.3c00076

**Published:** 2024-02-21

**Authors:** William R. Simpson, Jingqiu Mao, Gilberto J. Fochesatto, Kathy S. Law, Peter F. DeCarlo, Julia Schmale, Kerri A. Pratt, Steve R. Arnold, Jochen Stutz, Jack E. Dibb, Jessie M. Creamean, Rodney J. Weber, Brent J. Williams, Becky Alexander, Lu Hu, Robert J. Yokelson, Manabu Shiraiwa, Stefano Decesari, Cort Anastasio, Barbara D’Anna, Robert C. Gilliam, Athanasios Nenes, Jason M. St. Clair, Barbara Trost, James H. Flynn, Joel Savarino, Laura D. Conner, Nathan Kettle, Krista M. Heeringa, Sarah Albertin, Andrea Baccarini, Brice Barret, Michael A. Battaglia, Slimane Bekki, T.J. Brado, Natalie Brett, David Brus, James R. Campbell, Meeta Cesler-Maloney, Sol Cooperdock, Karolina Cysneiros de Carvalho, Hervé Delbarre, Paul J. DeMott, Conor J.S. Dennehy, Elsa Dieudonné, Kayane K. Dingilian, Antonio Donateo, Konstantinos M. Doulgeris, Kasey C. Edwards, Kathleen Fahey, Ting Fang, Fangzhou Guo, Laura M. D. Heinlein, Andrew L. Holen, Deanna Huff, Amna Ijaz, Sarah Johnson, Sukriti Kapur, Damien T. Ketcherside, Ezra Levin, Emily Lill, Allison R. Moon, Tatsuo Onishi, Gianluca Pappaccogli, Russell Perkins, Roman Pohorsky, Jean-Christophe Raut, Francois Ravetta, Tjarda Roberts, Ellis S. Robinson, Federico Scoto, Vanessa Selimovic, Michael O. Sunday, Brice Temime-Roussel, Xinxiu Tian, Judy Wu, Yuhan Yang

**Affiliations:** † Geophysical Institute, University of Alaska Fairbanks, Fairbanks, Alaska 99775, United States; ‡ Department of Chemistry and Biochemistry, University of Alaska Fairbanks, Fairbanks, Alaska 99775, United States; § Department of Atmospheric Sciences, College of Natural Science and Mathematics, University of Alaska Fairbanks, Fairbanks, Alaska 99775, United States; ∥ Sorbonne Université, UVSQ, CNRS, LATMOS, 75252 Paris, France; ⊥ Department of Environmental Health and Engineering, Johns Hopkins University, Baltimore, Maryland 21218, United States; ◆ Extreme Environments Research Laboratory, École Polytechnique Fédérale de Lausanne, EPFL Valais Wallis, 1951 Sion, Switzerland; □ Department of Chemistry, University of Michigan, Ann Arbor, Michigan 48109, United States; ■ Department of Earth and Environmental Sciences, University of Michigan, Ann Arbor, Michigan 48109, United States; ○ Institute for Climate and Atmospheric Science, School of Earth & Environment, University of Leeds, Leeds LS2 9JT, UK; ☼ UCLA Atmospheric & Oceanic Sciences, Los Angeles, California 90095, United States; ● ESRC/EOS, University of New Hampshire, Durham, New Hampshire 03824, United States; △ Department of Atmospheric Science, Colorado State University, Fort Collins, Colorado 80523, United States; ▲ School of Earth and Atmospheric Sciences, Georgia Institute of Technology, Atlanta, Georgia 30332, United States; ▽ Washington University in St. Louis, 1 Brookings Drive, Campus Box 1180, St. Louis, Missouri 63130, United States; ▼ Department of Soil, Water, and Climate, University of Minnesota, St. Paul, Minnesota 55108, United States; ⬡ Department of Atmospheric Sciences, University of Washington, Seattle, Washington 98195, United States; ⬢ Department of Chemistry and Biochemistry, University of Montana, Missoula, Montana 59812, United States; ◊ Department of Chemistry, University of California, Irvine, California 92697, United States; ⬠ Institute of Atmospheric Sciences and Climate (ISAC) of the National Research Council of Italy (CNR), Bologna 40121, Italy; ◇ Department of Land, Air, and Water Resources, University of California, Davis, California 95616, United States; ▰ Aix Marseille Univ, CNRS, LCE, 13331 Marseille, France; ☆ Office of Research and Development, U.S. EPA, Research Triangle Park, North Carolina 27709, United States; ★ Laboratory of Atmospheric Processes and their Impacts, Ecole Polytechnique Fédérale de Lausanne, 1015 Lausanne, Switzerland; ◁ Center for the Study of Air Quality and Climate Change, Foundation for Research and Technology Hellas, 26504 Patras, Greece; ◀ GESTAR-II, University of Maryland Baltimore County, Baltimore, Maryland 21250, United States; ▷ Alaska Department of Environmental Conservation, 555 Cordova St, Anchorage, Alaska 99501, United States; ▶ Earth & Atmospheric Sciences, University of Houston, Houston, Texas 77204, United States; $ IGE, Univ. Grenoble Alpes, CNRS, INRAE, IRD, Grenoble INP, 38000 Grenoble, France; % International Arctic Research Center, University of Alaska Fairbanks, Fairbanks, Alaska 99775, United States; ▱ Laboratoire d’Aérologie (LAERO), Université Toulouse III − Paul Sabatier, CNRS, 31400 Toulouse, France; & Alaska Department of Environmental Conservation, 610 University Ave., Fairbanks, Alaska 99709, United States; ¶ Finnish Meteorological Institute, Erik Palménin Aukio 1, P.O. Box 503, FI-00101 Helsinki, Finland; ⊡ Université du Littoral Côte d’Opale: Dunkerque, Hauts-de-France, 59375 Dunkerque, France; ⌽ National Renewable Energy Laboratory - Alaska Campus, Fairbanks, Alaska 99775, United States; ⊕ Institute of Atmospheric Sciences and Climate (ISAC) of the National Research Council of Italy (CNR), Lecce 73100, Italy; ⋈ Sustainable Energy and Environment Thrust, The Hong Kong University of Science and Technology (Guangzhou), Guangzhou, 511430, China; ⧓ Alaska Department of Environmental Conservation, P.O. Box 111800, Juneau, Alaska 99811-1800, United States; ⊖ Handix Scientific, Fort Collins, Colorado 80525, United States; ⟠ LMD/IPSL, ENS, Université PSL, École Polytechnique, Institut Polytechnique de Paris, Sorbonne Université, CNRS, 75005 Paris, France

**Keywords:** air pollution, aerosol particles, cold climate, atmospheric chemistry, Arctic, Alaska

## Abstract

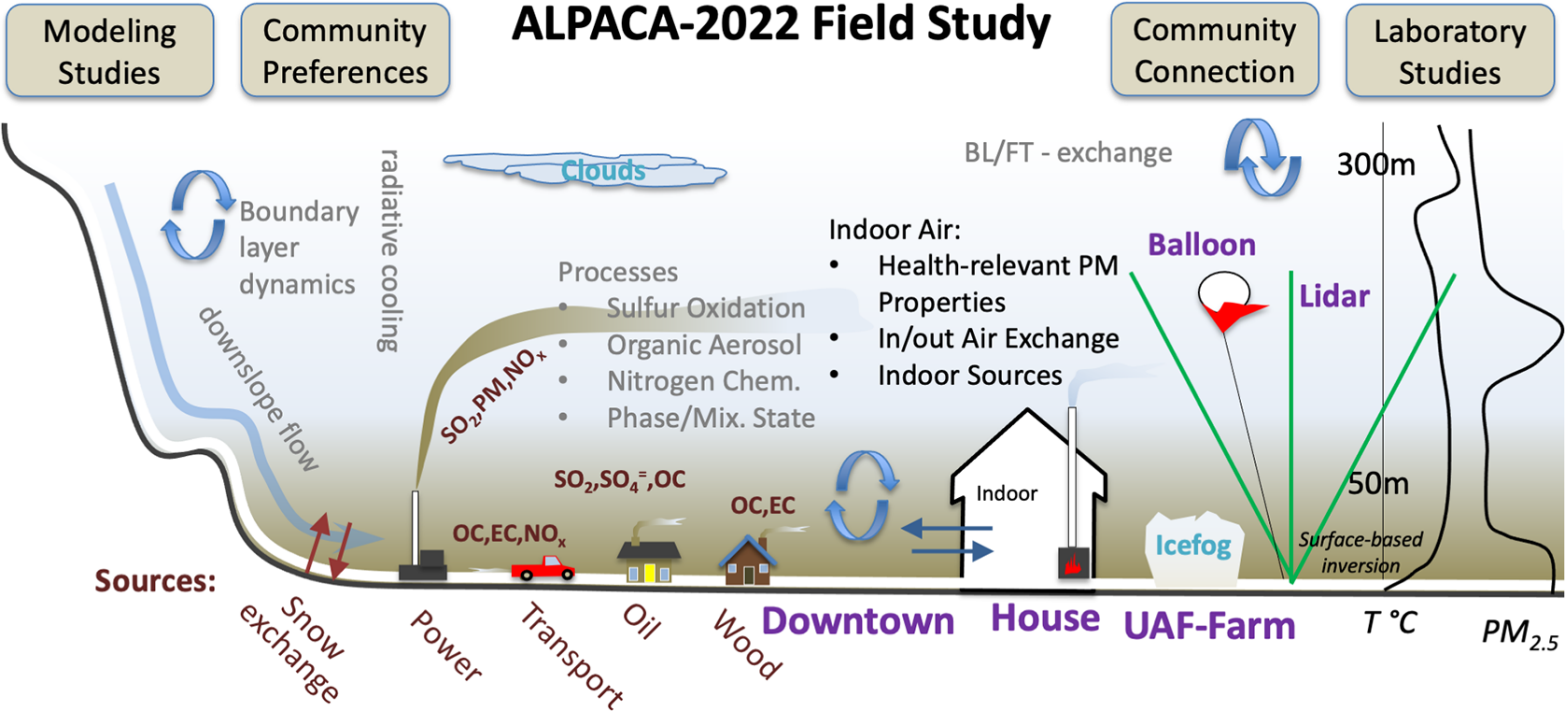

The Alaskan Layered Pollution And Chemical Analysis (ALPACA) field experiment was a collaborative study designed to improve understanding of pollution sources and chemical processes during winter (cold climate and low-photochemical activity), to investigate indoor pollution, and to study dispersion of pollution as affected by frequent temperature inversions. A number of the research goals were motivated by questions raised by residents of Fairbanks, Alaska, where the study was held. This paper describes the measurement strategies and the conditions encountered during the January and February 2022 field experiment, and reports early examples of how the measurements addressed research goals, particularly those of interest to the residents. Outdoor air measurements showed high concentrations of particulate matter and pollutant gases including volatile organic carbon species. During pollution events, low winds and extremely stable atmospheric conditions trapped pollution below 73 m, an extremely shallow vertical scale. Tethered-balloon-based measurements intercepted plumes aloft, which were associated with power plant point sources through transport modeling. Because cold climate residents spend much of their time indoors, the study included an indoor air quality component, where measurements were made inside and outside a house to study infiltration and indoor sources. In the absence of indoor activities such as cooking and/or heating with a pellet stove, indoor particulate matter concentrations were lower than outdoors; however, cooking and pellet stove burns often caused higher indoor particulate matter concentrations than outdoors. The mass-normalized particulate matter oxidative potential, a health-relevant property measured here by the reactivity with dithiothreiol, of indoor particles varied by source, with cooking particles having less oxidative potential per mass than pellet stove particles.

## Abstract 2

Air pollution in cold and dark regions is a critical understudied health problem. This overview reports on the design, implementation, and results from the Alaskan Layered Pollution And Chemical Analysis field experiment.

## Introduction

During the wintertime in cold regions such as the Arctic, poor dispersion of pollution, coupled with seasonally enhanced sources of pollution from heating, transportation, and industry, and unique chemical processing under cold and dark conditions cause high levels of local pollution.^1^ Residents of cold climate communities often ask questions about their wintertime air quality such as Which sources and processes dominate the pollution? How do weather and climate affect pollution? Is the air quality better or worse in my house than outside? These community concerns can be investigated by narrowing in on their underlying scientific questions: What are key sources of pollution in wintertime, and how do cold and dark conditions affect processing of pollution? How does cold-climate meteorology affect trapping of this pollution? How do outdoor air pollution and indoor sources affect indoor air quality? The international initiative PACES (air Pollution in the Arctic: Climate, Environment, and Societies),^2^ supported by the International Global Atmospheric Chemistry (IGAC) Project (under Future Earth) and the International Arctic Science Committee (IASC), recognized the need for coordinated, international, interdisciplinary research to study local Arctic air pollution sources and processes, while partnering with Northern communities. In May 2018, PACES, along with National Science Foundation (NSF) and National Oceanic and Atmospheric Administration (NOAA) support organized a workshop in Fairbanks, Alaska to identify key unresolved issues related to wintertime high-latitude urban air pollution. The workshop produced a white paper,^3^ which reviewed the history of this pollution and enumerated key open questions in this research area, leading to the ALPACA study described in this manuscript.

Recent studies, such as the Clean Air for London (ClearfLo) project,^4^ the Utah Winter Fine Particulate Study (UWFPS),^5,6^ Lake Michigan Air Directors Consortium (LADCO) Winter Nitrate study,^7^ Snow and Atmospheric Chemistry in Kalamazoo, Michigan (SNACK) campaign,^8−10^ and studies in Beijing, China,^11−14^ have investigated wintertime pollution chemistry in cities. These studies show that winter air pollution differs from that in summer, due to differing contributions of primary sources and altered chemical processes related to colder, darker, conditions, leading to distinct pollution characteristics affecting wintertime cities. A key source of high-latitude wintertime pollution in European and North American locations is wood smoke,^15−20^ which contributes mainly organic particles and gases.

Another important aspect of wintertime pollution is the meteorological influence, specifically the prevalence of temperature inversions, which reduce the vertical dispersion of pollution. This trapping of pollution can lead to unique chemical phenomena such as midwinter ozone production observed in the Uintah Basin in Utah.^21^ In that case, high VOC concentrations from oil and gas production activities led to carbonyl photolysis being a dominant oxidant source.^22,23^ Utah’s Salt Lake Valley experiences severe PM pollution episodes in winter that result from coupled trapping of pollution in persistent cold-air pools and pollution chemistry that leads to particulate nitrate formation.^24^ The Nitrogen Aerosol Composition and Halogens on a Tall Tower (NACHTT) study directly investigated the role of atmospheric vertical structure on chemistry.^25^ Airborne observations have also assisted in understanding regional aspects of wintertime chemistry, for example, in the Wintertime INvestigation of Transport, Emissions and Reactivity (WINTER) study in 2015.^26−28^ The recent perspective by Hallar and colleagues^29^ highlighted continuing knowledge gaps in understanding coupled chemical-meteorological processes during wintertime in mid-latitude basins and valleys with a focus on the western United States (U.S.), but with relevance to higher latitude cities.

It is generally recognized that people spend most of their day indoors, making indoor air pollution exposure a major concern, particularly in cold regions. Recent studies have investigated indoor air quality affected by indoor sources and interactions between indoor particles and gases. A key example of this type of study was the House Observations of Microbial and Environmental Chemistry (HOMECHEM) study.^30^ This study pioneered intensive collaborative multidisciplinary investigations into indoor air quality and provided a blueprint for the indoor component of the ALPACA field study. The U.S. National Academy of Sciences recently published a consensus study report on “why indoor chemistry matters”.^31^ These studies are leading to a growing literature of indoor pollutants, investigations into the role of indoor surfaces as reservoirs of pollution, and interactions between reactive species, such as ozone, and indoor gases, particles, and surfaces. Because other studies^30^ have investigated these aspects of indoor chemistry, we saw an opportunity in ALPACA to focus our indoor studies on wintertime-relevant indoor sources, such as wood heating, as well as the potential to probe indoor/outdoor air interactions across extreme indoor/outdoor temperature gradients.

Fairbanks, Alaska, is one of the most polluted cities in the U.S. during wintertime, regularly exceeding the Environmental Protection Agency (EPA) short term (24-h) outdoor standard of 35 μm m^–3^ for fine particulate matter (PM_2.5_, <2.5 μm in diameter) pollution. Past studies have highlighted the critical role of pollution trapping by temperature inversions in high pollution episodes.^32−36^ These temperature inversions can exceed 0.5 °C/m temperature gradient at the surface, greatly hindering vertical mixing.^37−40^ These same inversions trapped carbon monoxide (CO), causing Fairbanks to violate CO standard of 9 parts per million frequently up until the early 2000s,^41^ when better technology decreased their emissions from automotive sources. This source mitigation has been very successful, and now Fairbanks CO levels only reach about a third of the regulatory standard under the most severe inversion trapping conditions.

Fairbanks wintertime sources of particulate matter (PM) have been studied by chemical mass balance methods,^15^ which indicated that wood smoke sources caused 60–80% of ground-level PM from 2008–2011. Studies using positive matrix factorization (PMF) generally agreed that wood smoke was the largest single factor, but found lower percentages (40–52%) of influence in Fairbanks.^16,42^ This work, coupled with persistent wintertime PM_2.5_ violations, led to new regulations and incentives aimed at reducing pollution in general and wood smoke in particular. Specifically, a wood stove changeout program was implemented and, more recently, programs incentivizing the switch from wood or other fuels to cleaner-burning fuels has been operating. Air quality alerts, times when residents are not allowed to operate solid-fuel (*e.g.*, wood and pellet) stoves because of predicted poor dispersion of pollution, have been implemented.^43^ Note that waivers allowing wood burning during alerts are available for those who have no other source of heat or demonstrate good burning and wood storage practices. A recent PMF analysis found that the wood smoke contribution trended downward between 2013 and 2019,^44^ potentially in response to the interventions.

Although wood smoke has been the most targeted source in mitigation efforts, sulfate is the next most abundant component of PM, which has led to a recent focus on reducing sulfur emissions.^43^ Key sources of sulfur in the airshed are domestic heating oil and coal and diesel that fuel local power plants. Most residences in Fairbanks are heated by oil-fired boilers or furnaces, with wood and natural gas/propane as lesser contributors to home heat.^45^ The heating oils used in Fairbanks have a high sulfur content: ∼900 ppm S (parts per million of sulfur by mass) for #1 heating oil and ∼2500 ppm S for #2 heating oil.^46^ Most of the sulfur in these fuels forms SO_2_ upon combustion, contributing significantly to SO_2_ mixing ratios that average 10–15 nmol mol^–1^ in wintertime.^47^ A small fraction of the fuel sulfur is directly emitted as particulate sulfate or sulfur trioxide (SO_3(g)_) and rapidly hydrolyzes to form sulfuric acid, which soon forms PM or adds to pre-existing PM. Modeling studies^45,46^ of 2008 pollution episodes found that modeled sulfate was 34% of observed sulfate and noted that the model does not convert much SO_2_ to sulfate, suggesting that secondary sulfate is making up the difference. The mechanism of sulfur oxidation in this cold and dark (*e.g.*, low-photochemical activity) environment is not clear and is a major focus of the ALPACA field study. Modeling and observational studies have examined sulfur and nitrogen chemistry in Fairbanks.^35,48,49^ Recently, novel sulfur chemistry forming hydroxymethanesulfonate (HMS) during winter at sub-freezing temperatures has been observed in Fairbanks.^50^ HMS was originally discovered to be associated with fog^51^ and was recently observed in summertime fog at near-freezing temperatures in the Alaskan Arctic oil fields.^52^ During winter, HMS has been observed under polluted high-humidity (hazy) conditions in China.^53−55^ HMS formation contributes to PM sulfur in the S(IV) oxidation state and increases the PM mass concentration. Campbell and colleagues^50^ showed that HMS (S(IV) species) contributed 2.8% to 6.8% of PM mass during wintertime pollution episodes in Fairbanks. HMS may be a partial explanation for non-sulfate sulfur species measured in PM,^48^ and HMS could undergo further chemistry to oxidize to S(VI), providing a potential path to secondary sulfate.^53^ In addition, HMS and PM S(IV) species may be misidentified as sulfate either through fragmentation in aerosol mass spectrometer (AMS) instruments or through decomposition or co-elution in ion chromatography.^56^

## Experimental/Methods

### Study Goals and Design

**Figure 1:**
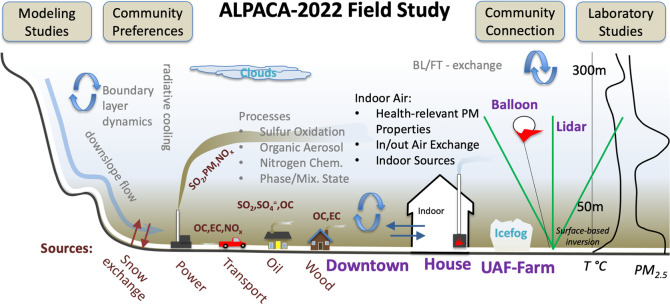
Schematic design of the ALPACA 2022 field study. Main questions and processes investigated are shown along with field sites. Boxes at the top represent broader ALPACA project activities connected with the field study. Community connections are outreach and education, while surveys were used to probe community preferences.

List 1 shows key goals of the ALPACA project. Figure 1 represents important processes and measurements made during the six-week ALPACA field study, which was carried out from January 17, 2022 through February 25, 2022. The broader ALPACA project also includes public engagement with the Fairbanks community, laboratory studies of processes, and modeling of Fairbanks air pollution. Detailed process studies and synthesis of the field study results will be the focus of future research and will be reported in subsequent manuscripts.

The motivation for the ALPACA project grew from community questions that arose from long-standing involvement of key research personnel in Fairbanks air quality issues.^57^ To further this conversation, the project began with community events (P1) in Fairbanks and the nearby city of North Pole in February 2020. Unfortunately, soon after those meetings COVID-mitigation restrictions reduced direct community connection. However, during the field study, we held virtual open houses to continue community connections, and after the field study, we published early results in the local newspaper. To understand how residents of Fairbanks and North Pole think about air pollution and wood burning and their preferences for solutions (P2), we mailed a survey to 3000 people in March and April 2022. Findings from the survey provided insights into the extent that perceived health and economic risks, trust in government, and affect (experience of feeling or emotion) relate to support for three different types of outdoor wood smoke mitigation policies in Alaska.^58^ To further community engagement, we partnered with a local teacher and worked with her to co-design a classroom-based citizen science program around air quality (P3). The program took the form of co-created citizen science, in which learners design and answer their own research questions. Students built sensors, designed questions, and collected and analyzed data. We used a pre-/postsurvey to ask about science identity-related outcomes among students.

List 1: ALPACA project goals:

**Public engagement goals**:

P1: Address community questions and share findings widely.

P2: Survey community attitudes on air quality and preferences for approaches to improve air quality.

P3: Engage community members in participatory research and study impact of this engagement.

**Atmospheric chemical goals:**

C1: Investigate how cold and low-photochemical conditions affect pollution.

C2: Quantify gas and particle chemical composition and apportion PM sources.

C3: Determine sulfur and nitrogen oxidation mechanisms including traditional photochemistry and non-traditional metal catalysis, brown carbon photochemistry, NO_2_, and other catalytic pathways.

C4: Study S(IV) and hydroxymethanesulfonate (HMS) abundance, formation mechanism, and fate in PM.

C5: Determine aerosol mixing state (distribution of chemical species within the aerosol population), as related to primary sources and chemical processing.

C6: Estimate PM acidity and study how it affects other chemical processes.

C7: Investigate what types of particles nucleate ice to form ice fog and how it alters particulate matter populations.

**Dispersion/transport goals:**

D1: Investigate vertical and horizontal dispersion of PM and trace gases under varying meteorological conditions.

D2: Determine the degree to which power plants affect ground-level breathing air quality.

D3: Examine how chemical processes vary as a function of altitude.

D4: Study processes influencing surface-based temperature inversions such as surface energy budgets, shallow cold flows, and advection of warm air above the surface cold pool.

D5: Determine the role of snow/air exchange and chemistry as a source and sink of gases and particles.

D6: Investigate the fate of air pollutants including deposition and potential impacts on climate compared to background Arctic haze

**Health relevant/indoor air goals:**

H1: Study health-relevant properties of indoor and outdoor wintertime urban pollution.

H2: Study indoor sources (*e.g.*, oil heaters, pellet stoves, cooking).

H3: Investigate infiltration of outdoor pollution and interactions with indoor sources.

H4: Study semivolatile partitioning, both outdoors and upon warming to indoor temperatures.

H5: Determine pollution sources and differences between residential neighborhoods and downtown.

Goals C1–C7 were addressed by co-locating instruments shown in Table 1 at the downtown CTC super site. State-of-the-art real-time particle^59−62^ and gas^62,63^ measurements probed interrelationships between these species (C1, C3, C4). Many of these instruments had minute to hourly time resolution, allowing us to observe plumes and improve knowledge of sources (C2). Measurements of PM ammonium^64^ and total (gas + particle) ammonia^65^ aided in understanding PM acidity (C6). To study PM S(IV) species (C4) and HMS, PM samples were briefly treated with hydrogen peroxide (H_2_O_2_), which converts non-HMS S(IV) species to sulfate but leaves most HMS as S(IV).^66−68^ Sampling of atmospheric particles onto substrates for off-line electron microscopy with energy-dispersive X-ray spectroscopy was carried out to assess aerosol chemical mixing state and coarse-mode particle sources^69,70^ (C5). Ice fog forms episodically under the cold temperatures and high humidity conditions of urban Fairbanks,^37^ typically on ice nucleating particles, likely affecting PM composition and chemistry. Therefore, goal C7 was capturing evolution in particulate matter composition, quantity, and ice nucleating properties during ice fog events, which were measured both via a continuous flow diffusion chamber^71^ in real time and offline^72^ from filters collected during the study.

**Table 1:** Instruments at Downtown Sites (CTC, NCore)^a^

measurement	instrument PI/Ref.
**Particulate Matter**	
Real-time nonrefractory PM composition via ACSM at Ncore	Weber, GT/Mao, UAF^126^
PM_2.5_, PM_10_ via BAM, PM_2.5_ speciation on 24-h filters	ADEC at NCORE
PM_2.5_ absorption at 7 wavelengths via Magee Scientific AE-33 Aethalometer	Mao, UAF
Real-time nonrefractory PM composition via HR-ToF-AMS	D’Anna/Temime-Roussel, LCE, France^59,62^
Particle organic composition via CHARON PTR-ToF-MS	
Nitrate multi-isotopic analysis (δ^15^N, δ^17^O, δ^18^O) via high-volume PM sampler, and aerosol composition	Albertin/Bekki/Savarino, LATMOS/IGE^127^
PM absorption and scattering (401, 870 nm; black carbon & brown carbon) via PAX	Yokelson, U. Montana^60^
PM absorption at 405, 515, 660, and 785 nm, via PAAS-4λ	Schnaiter, KIT, Germany^61^
Inorganic ion composition via PILS-IC	Weber, GT/Mao, UAF^64^
Total ammonia (soluble gas plus PM NH_4_^+^) via mist chamber	Weber, GT^65^
Size-resolved sulfate isotopes via high-volume PM sampler	Alexander, U. Washington^128^
Particulate mass and elemental composition via EDXRF	Creamean, CSU^129^
Particle Ice nucleation measurements, on and offline	Creamean, CSU^71,72^
Offline single-particle morphology and elemental composition via computer controlled-scanning electron microscopy with energy dispersive X-ray (CCSEM-EDX) spectroscopy of MOUDI samples (0.18–18 μm)	Pratt, UMich.^69,70^
Water-soluble ions in bulk aerosol (total PM) via filter/IC	Dibb, UNH^130^
PM size distributions, total PM number concentration, black carbon in PM_1_	D’Anna/Temime-Roussel, LCE, France
Size resolved water-soluble ions and metals via MOUDI and PM_2.5_ filters	Weber, GT^84^
Photooxidant production in PM_2.5_ from filters	Anastasio, UCD^131−133^
Particle water via wet/dry optical particle counters	Weber, GT^134^
**Gases**	
Volatile organic compounds/via CHARON PTR-ToF-MS	D’Anna, France^62^
Reactive gases: O_3_, SO_2_, CO, NO_*x*_	Simpson, UAF^36^
High precision CO	Hu, U. Montana^135^
NO_2_ multi-isotopic analysis (δ^15^N, δ^17^O, δ^18^O) via denuder gas sampling	Albertin/Bekki/Savarino, LATMOS/IGE^136^
Formaldehyde (HCHO) via COFFEE and Aeris instruments.	St. Clair, UMBC^63,137^
CO_2_, H_2_O via LiCor analyzer	Brus, FMI, Finland
**Meteorological Measurements**	
Temperature gradient (3m, 6m, 11m, 23m on CTC roof), winds on CTC roof	Simpson, UAF^36^
Photolysis rate measurements for NO_2_ and other gases via radiometers	Flynn, U. Houston
Wind Lidar profiling (first half of study)	Dieudonné, LPCA/UCLO, France
**Snow sampling**	
Snow ionic composition, surface and pits	Dibb, UNH^138^
Nitrate multi-isotopic analysis (δ^15^N, δ^17^O, δ^18^O) in snow samples	Albertin/Bekki/Savarino, LATMOS/IGE, France
Ionic composition, metals, and organic tracers in snow samples	Scoto, Italy^75,76^

a The reference is for the technique used.

Goals D1–D6 were addressed at several locations around Fairbanks via measurements listed in Table 2. Dispersion of pollution (D1, D2) was studied by vertically resolved measurements on buildings,^36^ via remote sensing,^73^ and using tethered balloons.^74^ Surface-based temperature inversions (D4) and their role in boundary layer structure were addressed by vertical profile and radiative flux measurements and through modeling. Snowpack exchange (D5) was investigated by sampling surface snow^75,76^ both downtown and at the University of Alaska Fairbanks UAF-Farm. Understanding the fate of exported pollution from Fairbanks and import of Arctic pollution such as Arctic haze into Fairbanks (D6) was investigated by measurements at research sites of varying distance from downtown. Many modeling studies are planned to understand coupled chemistry/transport (D1–D6). The extremely strong near-surface temperature inversion and low speed winds at the surface require a fine vertical grid to model the trapping of pollution from surface sources and also to model potential downwash from lofted sources such as power plants. Therefore, we used both 1-D chemistry-aerosol-transport models^49,77,78^ with very fine grids and a special version of the Weather Research Forecast - Community Multiscale Air Quality (WRF-CMAQ) model tuned to Fairbanks^46^ and coupled with aerosol chemistry and WRF-Chem tuned to Arctic conditions.^79^ To model dispersion, we can couple meteorological fields from WRF with a Lagrangian model such as the FLEXible PARTicle dispersion model (FLEXPART).^80^ Chemical-aerosol mechanisms in these models are likely to need improvements to deal with new chemistry related to goals (C1, C3, and C4) and also with chemical effects of aerosol mixing state (C5), acidity (C6), and ice/super-cooled liquid phase (C7).

**Table 2:** Vertical Profile Measurement Instrumentation^a^

measurement	instrument PI/Ref.	location
**Downtown Vertical gas measurements**		
Vertical distribution of O_3_, NO_2_, SO_2_, HCHO, and HONO downtown to Birch Hill (∼200 m above valley floor) via LP-DOAS	Stutz, UCLA^73^	DOAS Base/hill
Birch Hill Ski Hut, in-situ ozone measurement using Teledyne 400E, 158 m above valley floor	Simpson, UAF	Birch Hill
Gradients via low-cost gas sensor: NO, NO_2_, CO, O_3_, & PM (3 m and 20 m on CTC roof)	Roberts, LPC2E, France	CTC
CO_2_ gradient (3 m and 23 m on CTC roof)	Simpson, UAF^36^	CTC
**Swiss tethered balloon, Helikite**		
Swiss Helikite balloon payload including RH, T, wind speed, pressure, PM size and number via OPC, mini SMPS, and CPC, PM light absorption via STAP, offline sampling, CO_2_, O_3_, CO	Schmale, EPFL, Switzerland^74^	UAF-Farm
CNR payload: VOCs gas sensor for benzene, toluene, ethylbenzene, and xylene	Decesari, CNR, Italy	UAF-Farm
MicroMegas balloon payload: O_3_, CO, NO, NO_2_	Barret, LAERO, France	UAF-Farm
**Meteorological Measurements**		
Surface turbulent fluxes via eddy covariance, radiation, RH, T, scintillometer, acoustic sounder, microwave radiometer	Fochesatto, UAF	UAF-Farm
Wind Lidar profiling (CTC for first half of study, UAF-Farm for second half of study)	Dieudonné,^90^ LPCA/UCLO, France	CTC/UAF-Farm
Meteorological station including Wind direction and speed, radiation, RH, T	Schmale, EPFL, Switzerland	UAF-Farm
Shortwave and longwave radiative fluxes, winds, and temperatures on a 10m tower	Ravetta, LATMOS, France	Goldstream Valley
**Particulate Matter and Trace Gases**		
Meteorological and particle turbulent fluxes via eddy covariance	Donateo, CNR, Italy^139^	UAF-Farm
Aromatic VOC, organic molecular tracers, metals, ionic composition, oxidative potential via DTT	Decesari, CNR, Italy	UAF-Farm
Particle size distributions via SMPS and OPC, PM composition via MOUDI, ozone, CO	Schmale, EPFL, Switzerland	UAF-Farm
**Snow sampling**		
Nitrate multi-isotopic analysis (δ^15^N, δ^17^O, δ^18^O) & aerosol composition in snow samples	Albertin/Bekki/Savarino, LATMOS/IGE, France^127^	UAF-Farm
Ionic composition, metals, and organic tracers in snow samples	Scoto, Italy^75,76^	UAF-Farm
**Regional background air composition**		
Nitrate and sulfate isotopes via high-volume PM sampler, ionic composition and nitrate isotopes in snow, and PM size distribution	Savarino/Albertin/Bekki, LATMOS/IGE, France	Poker Flat
Carbon monoxide and black carbon long term measurements, Kanaya, JAMSTEC, Japan	Kanaya, JAMSTEC, Japan	Poker Flat

a The reference is for the technique used.

We investigated indoor and outdoor air quality at a house in a residential neighborhood near downtown to pursue goals H1–H5. Again, a suite of state-of-the-art real-time particle^59,81,82^ and gas^83^ measurements were carried out, as listed in Table 3. The neighborhood surrounding the house had different sources from the more urban downtown site; therefore, the contrast between measurements of outdoor air at the house and downtown contributes to improving understanding of sources and chemical processing (C2, C4, C5, H5). Measurements of health-relevant properties, such as the oxidative potential (OP),^84−87^ of PM both indoors and outdoors were used to address goals H1 and H2. Infiltration and changes to particles upon warming (H3, H4) were investigated by alternately sampling indoor and outdoor air with these real-time instruments and comparing gas and particle composition, including analysis by a semivolatile gas/particle (SV-TAG) instrument.^88,89^ The SV-TAG, the single-particle aerosol time of flight mass spectrometer (ATOFMS), and the high-resolution aerosol mass spectrometer (HR-AMS) can detect chemical species such as polycyclic aromatic hydrocarbons (PAHs) and quinones that are known to affect OP, so the combination of these measurements should improve understanding of molecular species involved in outdoor and indoor health-related properties such as OP.

**Table 3:** Instruments at the Research House^a^

measurement	instrument PI/Ref.	inlet
**Particulate Matter**		
Real-time nonrefractory PM composition via HR-ToF-AMS, PM black and brown carbon	DeCarlo, JHU^59^	10 min in/10 min out
Semivolatile organic aerosol particles and gases via SV-TAG	Williams, WUStL^88,89^	1 h in/1h out
Real-time single-particle composition via ATOFMS	Pratt, UMich^81,82^	10 min in/10 min out
Aerosol size distributions via SMPS and APS	Pratt and DeCarlo^140^	10 min in/10 min out
Nanocluster aerosols via CPC, size via SMPS, and fluorescent particles via WIBS	Licina, EPFL, Switzerland	inside
Outdoor bulk PM sampling for environmentally persistent free radicals (EPFRs) and reactive oxygen species (ROS) via EPR	Shiraiwa, UCI^86^	outside
Indoor size-resolved PM sampling via MOUDI	Shiraiwa, UCI^87^	inside
Indoor PM_2.5_ filter oxidative potential (OP) via DTT assay	Weber, GATech^85^	inside
Outdoor PM_2.5_ and size-resolved PM OP via DTT assay	Weber, GATech^84^	outside
Indoor and outdoor low-cost optical PM sensors	DeCarlo, JHU	in/out
Photooxidant production in PM_2.5_ from filters	Anastasio, UCD^131−133^	outside
**Gases**		
Volatile organic carbon gases (VOCs) via PTR-ToF-MS	Hu, U. Montana^83^	10 min in/10 min out
Reactive Gases: O_3_, NO, NO_2_, NO_*x*_, NH_3_, HCHO	Yokelson, U. Montana,^60^ DeCarlo, JHU	10 min in/10 min out
Greenhouse Gases CO_2_, CH_4_, CO, H_2_O	DeCarlo, JHU	10 min in/10 min out

a The reference is for the technique used.

### Downtown Sites

**Figure 2:**
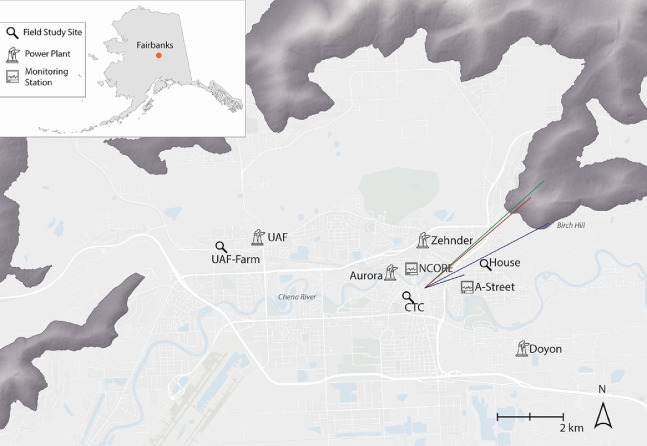
Map of Fairbanks, Alaska showing key field sites, power plants, and ADEC air monitoring stations used in the ALPACA 2022 study. See text for geolocation. The dark shading shows hills surrounding Fairbanks, and the lines from downtown to Birch Hill are the LP-DOAS light paths.

Figure 2 shows the locations of the field sites. Most of the downtown effort was focused on the University of Alaska Fairbanks Community and Technical College (CTC) site, where gas and particle sensing instruments (Table 1) were located in two trailers parked at the base of the CTC building (64.841°N,147.727°W, 135 m AMSL). Instruments in these trailers generally sampled from independent inlets, chosen to be compatible with the analyte of interest, that were secured to roofs of the trailers and sampled between 3 and 4 m above the ground level (AGL). The site was surrounded by a perimeter fence, and several filter samplers and other instruments were placed on the ground within the perimeter.

The CTC air sampling site is adjacent to a major downtown road, Barnette Street (∼40 m horizontal distance), and along the less busy 7th Avenue (10–20 m distance), which had on-road parking. Across Barnette Street is the Fairbanks State Office Building (SOB), which was the main downtown air quality monitoring site before the National Core (NCore) monitoring site was established in 2009 and was determined to have equivalent pollution levels, allowing SOB to be shut down in 2019. The NCore site, operated by the Alaska Department of Environmental Conservation (ADEC) for the U.S. EPA, is 580 m north of the CTC site and provides a long-term context for these downtown measurements. To study air-snow interactions, surface snow and episodic snow pits were sampled at the NCore site, and in-snow temperature and light profiles were recorded.

These downtown sites are in the urban core of Fairbanks. To the north and east of the site are mostly commercial businesses that are active during business hours. Commuting to these businesses leads to significant rush-hour traffic signals. However, to the west of this site, one to two story residential homes dominate, so it has a mix of sources. Figure 2 shows two coal-fired power plants are near CTC: the Aurora Energy power plant lies 900 m to the northwest of this site and the Doyon Utilities Ft. Wainwright Combined Heat and Power Plant lies 4 km to the southeast of this site. These two power plants have moderately high stacks (48 and 24 m, respectively) designed to reduce ground-level pollution, but a major effort of our study is to determine this strategy’s effectiveness. The Zehnder power plant employs a diesel-fired generator with a relatively low stack (18 m) and is located 1.5 km NNE from CTC, operating periodically to cover electrical grid load variations. Emissions estimates from these power plants, transportation, home heating, and other sources are available from ADEC.^46^ The Aurora Energy plant provides steam heat to a region of downtown buildings including the CTC building and others mostly to the north and east of CTC. This heat reduces pollution from the typical heating fuels, oil, wood, and gas and makes this site a contrast to the more residential “house” site (described below) chosen for indoor/outdoor studies.

### Vertical Profiling Sites

Goals D1–D6 are addressed by observing the vertical structure of pollution using methods in Table 2, which were carried out at multiple sites. At the CTC site, an 11 m tower was outfitted with temperature and chemical sensors, and another set of sensors was placed on the roof of the building at 23 m AGL to probe the role of the near-surface temperature inversion in trapping pollution. A Doppler wind lidar (Vaisala WindCube v2),^90^ which probed wind fields 40–300 m AGL, was located at the CTC site for the first half of the study. UCLA’s long-path differential optical absorption spectrometer (LP-DOAS) used spectroscopy to measure path-averaged pollutant concentrations (O_3_, NO_2_, SO_2_, HCHO, and HONO) from its base in a parking garage at 64.844°N, 147.716°W, which is 610 m ENE of the CTC building. The LP-DOAS measured to the ENE direction over the house research site (described below) along four paths sampling the vertical distribution of pollution between 12 and 191 m above the valley floor, with approximate path locations indicated on Figure 2. Three of these reflectors were on Birch Hill, at 73, 115, and 191 m above the valley floor and were about 4 km from the LP-DOAS base. The lowest path was nearly parallel to the ground and viewed a reflector on the roof of Nordale Elementary School (64.847°N, 147.693°W), 12 m above the valley floor and 1.15 km from the base. Near the summit of Birch Hill, at the cross-country ski facility (64.869°N, 147.648°W, 293 m AMSL, 158 m above the valley floor), an in-situ ozone monitor probed air aloft.

The above sites were picked to be near the downtown site and the house site to sample pollution related to urban pollution problems and surrounding near-downtown residential neighborhoods. To sample air further aloft, a tethered balloon was deployed in a large open farm field to the west of downtown. This UAF-Farm site (64.853°N, 147.860°W) is 6.5 km WNW from CTC and subject to traffic, railroad, residential, and possibly airport emissions. The UAF-Farm site has been the site of meteorological studies in the past,^38,39^ and a meteorologically focused overview of studies carried out at that site is being prepared for separate publication (Fochesatto et al., in preparation). Therefore, we mostly focused on the particle and gas monitoring done at that site. A key measurement platform deployed at the UAF-Farm site was the Swiss (EPFL) tethered balloon sampling system.^74^ This platform carried particle and gas sampling equipment from the surface to 350 m AGL, allowing the system to probe both surface polluted layers and lofted layers sourced by power plants. A 10 m tower measuring PM abundance and turbulence was installed to investigate PM deposition fluxes. During the second half of the study, the Doppler wind lidar was moved from CTC to the UAF-Farm site. Background air composition (see Table 2), including Arctic haze, and meteorological parameters were sampled 34 km northeast of downtown Fairbanks at the hilltop site at Poker Flat Research Range (65.118°N, 147.433°W, 502 m AMSL).

### House Site

To study the influence of both outdoor air and indoor sources on indoor air quality, we rented a house in the Shannon Park Neighborhood of NE Fairbanks. This house (64.850°N, 147.676°W) was located 2.6 km ENE from CTC and about 1 meter above the CTC site altitude. Most of the downtown urbanized area of Fairbanks is within a few meters vertically of the level of the Chena River, which flows through town. The house site was picked to be near the ADEC A-Street site, a pollution-impacted residential neighborhood site, which is 800 m WSW of the house and 1.8 km ENE of CTC. The A-Street site is in the Hamilton Acres neighborhood, which is directly adjacent to Shannon Park, with the house being about 2 blocks from the border.

**Figure 3:**
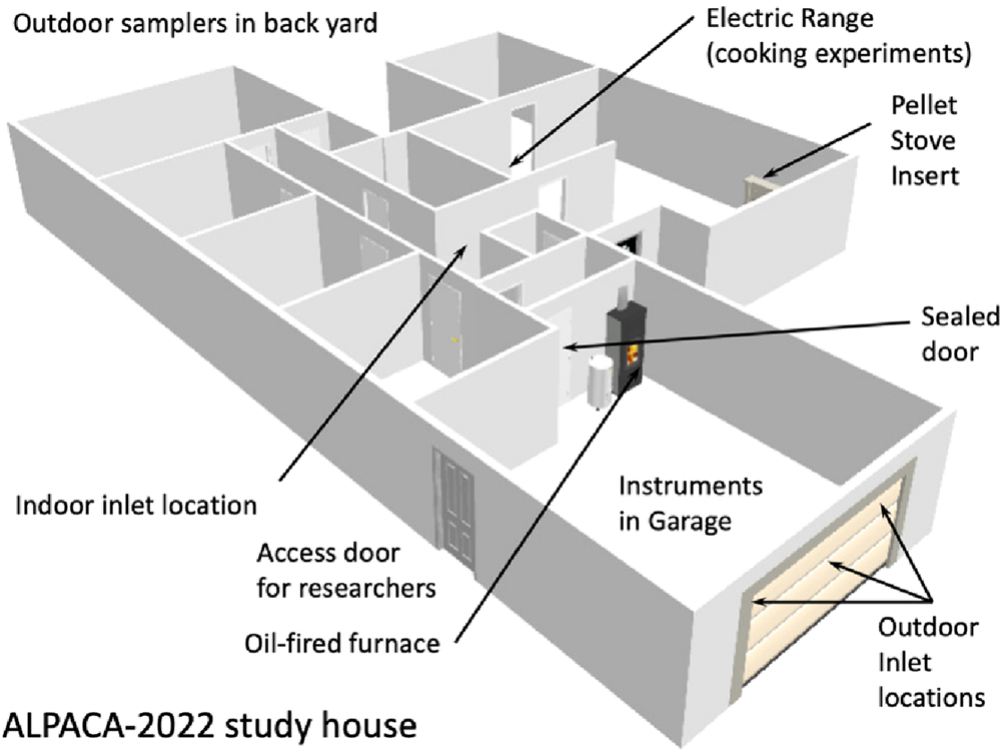
A 3-D rendering of the house’s plan showing locations of inlets (in the main house and outside the garage) and key indoor sources (cook stove, pellet stove). As discussed in the text, most instruments were located in the garage and automated switching valves alternately sampled from indoor or outdoor inlets.

The single-story house had a footprint of 1549 square feet (144 m^2^) and an attached garage of 531 square feet (49 m^2^). Most of the instruments (see Table 3) were in the garage, which was isolated from the main house. Figure 3 shows a model of the rooms in the house, the locations of the indoor and outdoor sampling ports, and indoor potential pollution sources. Many instruments used indoor/outdoor switching inlets,^91,92^ while some instruments were physically located in the house, or on the outdoor back porch of the house, as described in Table 3 and Figure 3.

The house site was in a residential neighborhood, with typical lot sizes of approximately 1/4 acre (∼1000 m^2^). The extreme cold climate of Fairbanks leads to high home heating costs, large greenhouse gas emissions,^93^ and potentially to high emissions of pollutants, depending upon the heating source’s emissions per heat delivered. Surrounding houses likely had a typical mix of fuels used in Fairbanks residences, which are dominated by heating oil (the primary heating source used in our test house), with some use of wood heat.^45^ Recently, natural gas supply in Fairbanks has increased, and some houses in the neighborhood have converted to gas. Compared to the downtown sites (CTC and NCore), there would be less impact from mobile sources because of the lack of major road arteries in the neighborhood, and a generally more “residential” mix of pollution. The house was about 350 m from the base of Birch Hill, which abruptly rises at the eastern edge of Fairbanks. The absence of residences to the east of this edge and much greater population to the west probably contributes to the gradient across this region and the Shannon Park neighborhood having lower outdoor pollution levels than at A-Street (Hamilton Acres) or the downtown sites.^94^

### House Methods

Before the start of the field study, an energy audit was conducted on the house to determine air tightness and thermal resistance. A housing ventilation researcher from the National Renewable Energy Laboratory’s Alaska Campus carried out these tests and translated the results to the standard energy rating used in residential sales in Fairbanks, which is a “star” rating from 1 to 6 stars. The house rated just above the border between 3+ and 4 stars, representing a slightly above average energy rating compared to the local housing market. The audit included a blower door test where the house was depressurized by 50 Pa, and envelope leakage air flow was measured to be 2.6 air exchanges per hour. Based upon this leakage at 50 Pa, 0.12 air exchanges per hour were estimated for an annual average under natural conditions and 0.19 air exchanges per hour under design winter conditions. However, the residence time of pollution in the house is a function of many more variables, such as thermal buoyancy of the warmer air in the house and winds on the outside of the house, which cause pressure gradients. Although this house has no active heating recovery ventilation system, many local homes do, and, if present, these systems also affect indoor pollution residence times. After instruments were installed and the field study was operational, a second blower door test re-measured the house tightness in the experimental configuration, finding slightly higher leakage of 2.8 air exchanges per hour at 50 Pa pressure difference. The house was heated by a hot-air furnace system, and the furnace air recirculation fan was switched to continual operation to keep the house mixed.

A key goal of the study (H2) was to investigate indoor pollution sources relevant to cold climate regions. Therefore, we installed a pellet stove insert (Harman P35i) in the house’s open fireplace to investigate potential influences on indoor air quality from operating this stove. The stove was professionally installed, and a “Stage 1” research waiver was obtained from ADEC to allow the team to operate the stove in moderately polluted conditions. The house had a smooth-top “ceramic” electric range that was used for cooking experiments.

Most instruments were placed in the attached garage, and the door between the garage and the house was replaced and sealed to separate the house from the instruments. The external garage door was also replaced so that inlets could be routed through the replacement temporary wall to sample outside air. Table 3 lists strategies for sampling indoor/outdoor air in the “inlet” column. Automated switching valves were installed to alternately sample from inlets drawing air from the house or from outside the garage. The first inlet and automated valve system^91^ used a 10 min indoor/10 min outdoor cycle and delivered air through a Nafion dryer to Johns Hopkins University (JHU) and U. Michigan instruments. A second inlet and automated valve, also operating on a 10 min in/10 min out cycle delivered air to the U. Montana PTR-MS and gas monitors. Both fast (10 min) switching valve systems used a bypass to maintain continual flow for both the sample and unsampled inlets as well as thermal insulation around the lines to maintain ambient environmental conditions in the lines. The tubing from the switching valve to the instruments was kept as short as possible. Due to slower sample analysis time, the Washington U. St. Louis SV-TAG instrument required a 1 h indoor/1 h outdoor cycle and used its own switching valve system.^92^ For the SV-TAG measurements of gas/particle partitioning, extensive care was taken to maintain the equilibrium between the gas and particle phase at the temperature of the outdoor/indoor environment being sampled. The particle phase was sampled by removing the gas phase on two different denuders, which were maintained at either outdoor or indoor temperature. Losses of gases and particles on walls of inlet tubes were minimized by conditioning the lines with continual flow of air of the next sample type between ambient injections.

Indoor experimental perturbations included heating with the pellet stove, simple cooking activities, and burning incense. To test for interactions between these indoor sourced particles and gases with each other and with infiltrated outdoor particles, “mixed” experiments with multiple activities were also performed. Although people affect indoor chemistry,^95,96^ we felt that there would not be enough time to include the effects of human occupation with sufficient replication and therefore decided to minimize household occupation. When there were no experiments or other necessary activities, the house was unoccupied. This strategy allowed for long periods, particularly at night, when infiltration of air into the house was expected to be the dominant source of particulate matter.

## Results and Discussion

### Environmental and Pollution Conditions Encountered

A number of unusual conditions occurred during the ALPACA field study. The COVID-19 pandemic reached the peak in the Fairbanks North Star Borough during this period, with a daily average new case count of more than 350 cases/100,000 residents (in late January 2022), although the peak in hospitalizations happened in the prior Fall. It is unclear how COVID-19 mitigation or pandemic-induced community choices may have affected pollution emissions. In late December 2021, just before the field study, the area experienced a series of warm winter storms that dumped freezing rain on sub-freezing roads and prior snowpack.^97^ This rain formed a thick ice layer in the snowpack and coated roads with a few cm of hard ice. Subsequent snow buried the ice layer in the snow pack, but the ice is likely to have restricted air motion in the snowpack, potentially changing deposition or snow chemical processes. During the rain-on-snow event, driving was certainly reduced, but by the time the campaign started, most driving activities appeared to return to near normal levels. The ice on the roads persisted in many places for much of the study’s duration, and plowing/snow removal activities were probably more prevalent than normal. Although these events were certainly unprecedented, winter weather in Fairbanks and high latitude cities in general is highly variable in terms of temperature, snowfall, ice, and other environmental variables because storms rather than daily solar heating are the main source of heat and moisture.

**Figure 4:**
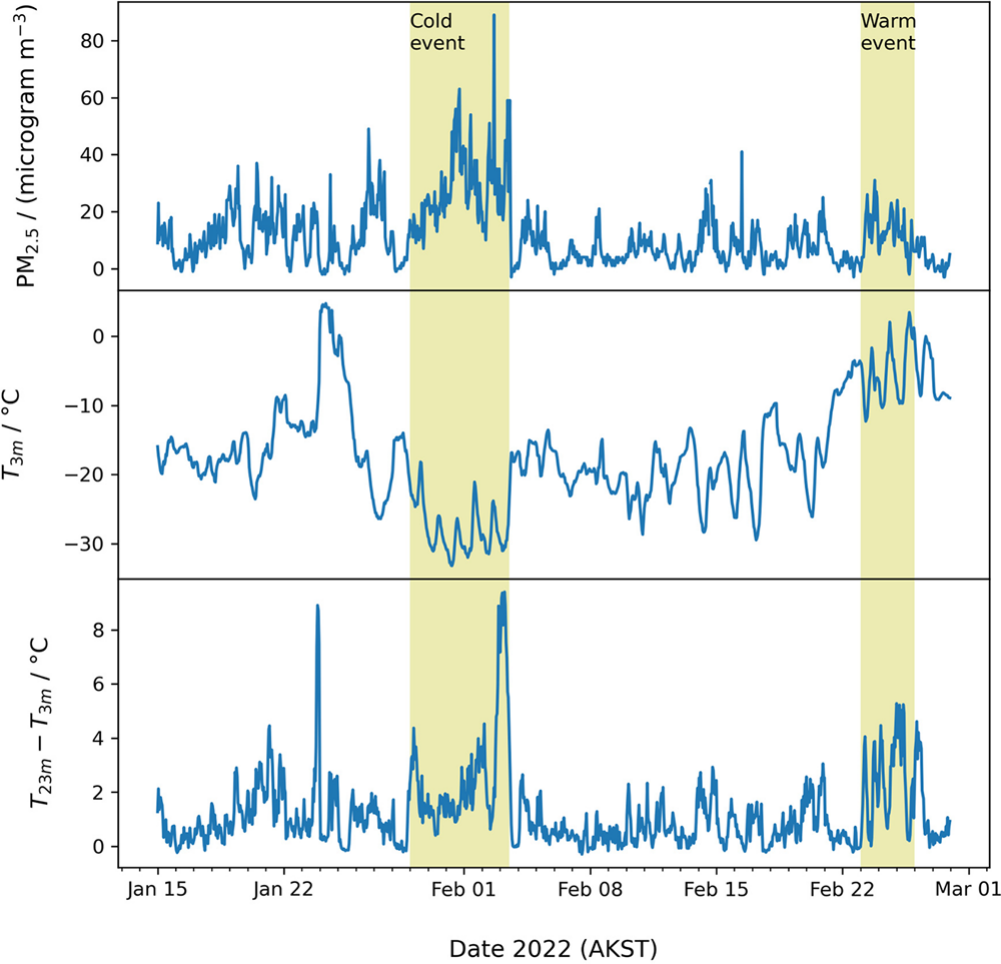
Hourly particulate pollution (PM_2.5_) measured at NCore by ADEC, temperature at 3 m AGL, and temperature inversion strength, represented by a temperature difference between 23 m and 3 m (temperatures measured at CTC), encountered during the ALPACA 2022 field study. Contrasting cold and warm pollution episodes are identified as discussed in the text.

Figure 4 shows pollution and temperature data during the ALPACA field study. The sunlit length of day varied from about 5.3 h (January 17) to 9.7 h (February 25). Hourly PM_2.5_ concentrations during the study varied up to about 80 micrograms m^–3^, as measured by the ADEC beta attenuation monitor at NCore. A measurement of the surface-based inversion (SBI) strength is the temperature difference between the top of the CTC building (23 m AGL) and the ground-level sensor (3 m), shown on the lower panel. Two meteorological mechanisms commonly form these temperature differences.^98−102^ First, radiation cooling at the surface at night can reduce the surface temperature, while having less cooling effect aloft, forming a temperature inversion. A second mechanism occurs when warm air from outside Fairbanks advects towards the area, flowing above the cold pool of air trapped by the hills surrounding Fairbanks. Clear sky periods allow strong infrared radiation cooling driven SBIs, which typically happen diurnally at night. In December and January, the sunlit period is short, and the albedo is high enough to limit daytime warming such that SBIs can persist over multiple days. However, in February and March, increasing daytime insolation often cause SBIs to break in the afternoon, and SBIs start to follow a stronger diurnal cycle.

During the field study, researchers noted two periods of extreme conditions that had enhanced pollution. The first of these, which we highlight as the “cold pollution event” spanned January 29 until the early afternoon of February 3. This event had some of the coldest conditions of the study period, with temperatures of −20 to −35 °C, and some patchy ice fog was noted. During these periods, many groups increased filter collection sampling frequency to capture finer details and/or day/night differences. Particulate pollution spiked to the campaign high. A second event, the “warm pollution event”, occurred at the end of the campaign, from February 23 to February 25. This event was much warmer, −12 °C to above freezing, but pollution was significantly elevated, with PM_2.5_ exceeding 30 micrograms m^–3^. Nighttime temperature inversions were quite strong in this period, likely indicating significant trapping of pollution. We point these events out because they show that pollution occurs at a wide range of temperatures and environmental conditions, and future manuscripts are likely to consider these periods.

**Figure 5:**
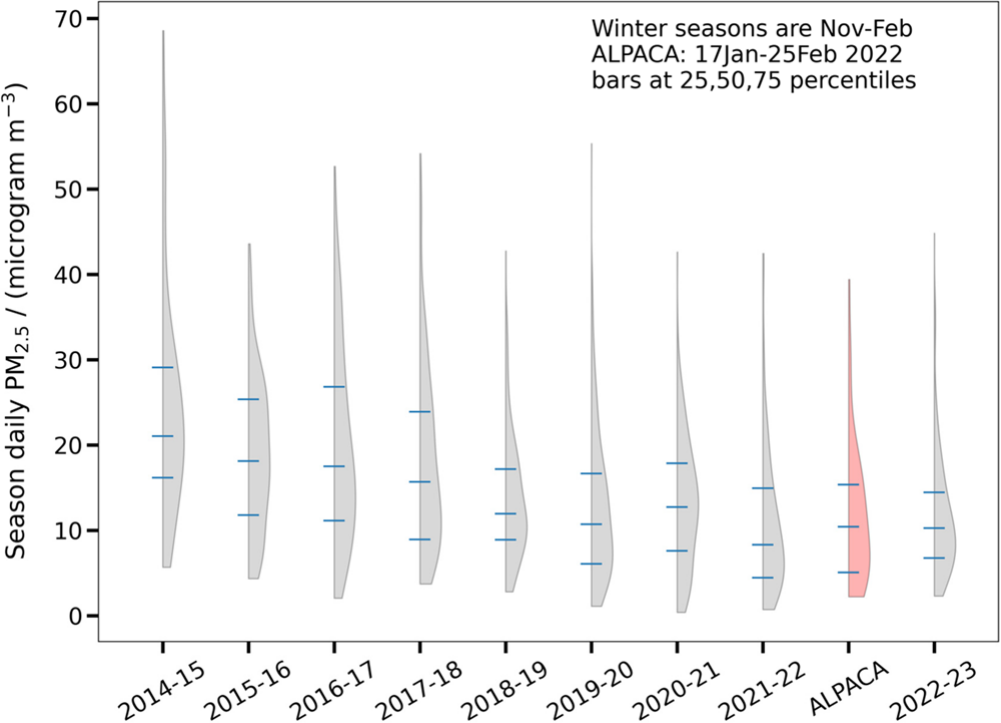
Probability density distributions of daily average particulate matter concentrations (PM_2.5_) from NCore (ADEC/EPA measurements) during recent winters (November of the first year to end of February of the second year listed). The red figure shows the distribution during the ALPACA field study, a subset of the 2021–2022 winter season’s data. Bars within each distribution represent the first quartile, median, and third quartile of the distribution.

Wintertime conditions and pollution levels clearly varied greatly in this study period, so we wanted to see how they compared to recent wintertime conditions. Figure 5 shows violin plots (probability densities) for daily PM_2.5_ concentrations in winter seasons from November to the end of February. This figure demonstrates that from about 2014 to 2018, pollution levels decreased, with less evidence of change after 2018. The ALPACA field study period is shown as a red-shaded distribution, and this period appears to be within the envelope of recent variability indicating that our study encountered pollution conditions typical of recent winters, at least with respect to PM concentrations.

### Results in Response to Community Questions

Some researchers on the ALPACA team have been involved in air quality studies for decades, including involvement in the 2018 Air Quality Stakeholders Group,^57^ which had the mission “to identify, evaluate, and recommend community-based solutions to bring the area into compliance with federal air quality standards for fine particulates.” During the stakeholders group process and at ALPACA open houses, it became clear that the community had many questions, which often were not directly related to outdoor air quality regulations and therefore were not being addressed. Therefore, we designed the study to address a broad spectrum of community concerns. In the sections below, we highlight how the study is providing first insights into these questions in a series of short vignettes. Subsequent manuscripts will expand upon these ideas, address others, and use modeling and laboratory studies to further improve mechanistic understanding of key processes and to evaluate the implications of the study results. The ALPACA project also surveyed the community to understand preferences and attitudes towards air quality problems, which will be reported upon in the peer-reviewed literature. Continuing community connection and discussions with regulators are also underway.

### Infiltration of Outdoor Air into a Residence

Fairbanks community members often point out that outdoor air pollution is measured, but they spend nearly all of their time indoors because of the cold climate. Therefore, they ask “How is the air quality in my house?” This is a complex question that we can break down into a series of sub-questions, the first of which would be how much outdoor pollution infiltrates into a house. Because we understand that indoor activities and temperature-dependent volatilization will affect many PM species, we decided to explore the indoor/outdoor ratio of pollution for sulfate, a nonvolatile PM component dominantly originating outdoors.

**Figure 6:**
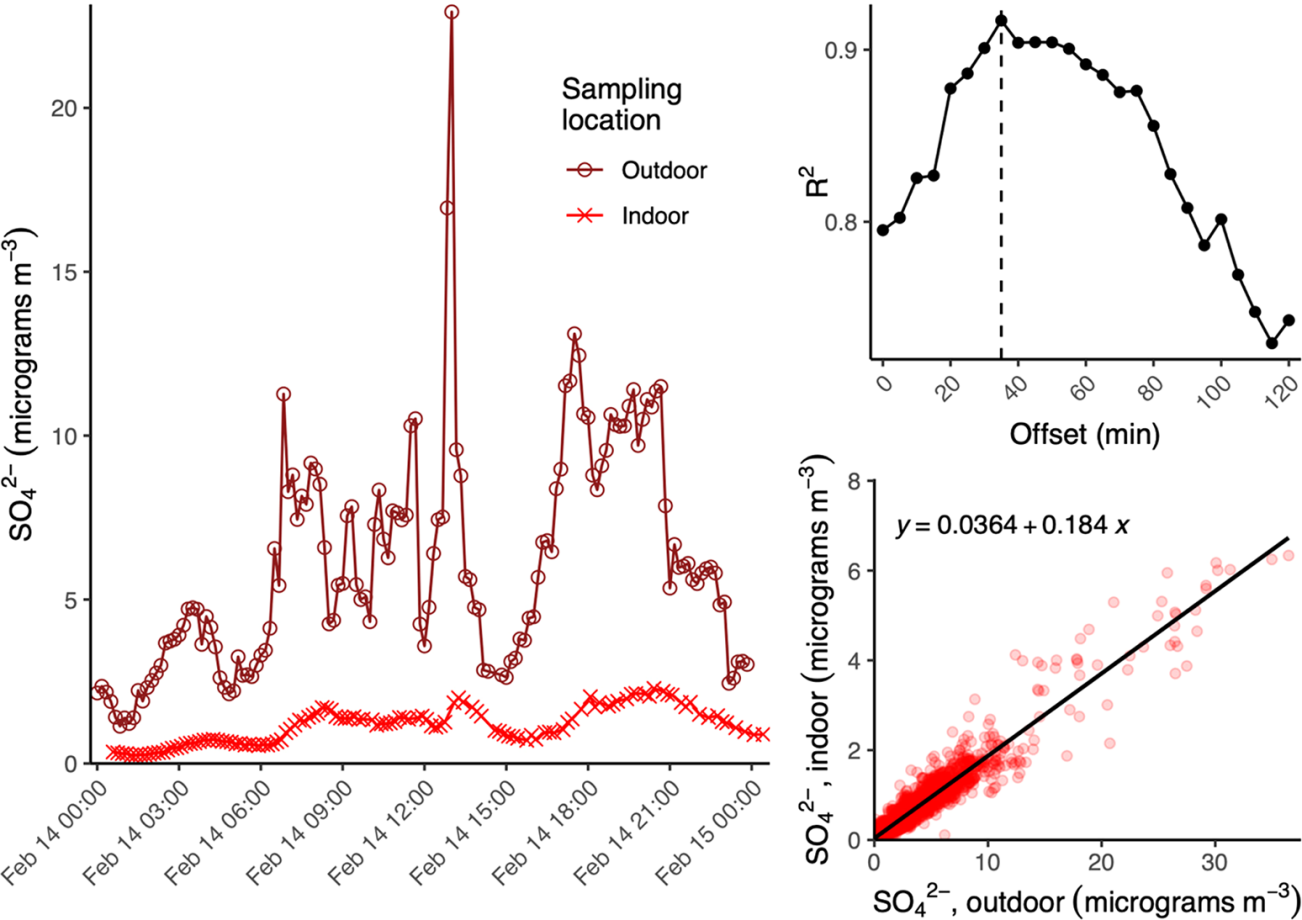
Indoor and outdoor PM sulfate as measured by the JHU HR-AMS at the house site. Date times are in AKST. The left panel shows the time series of sulfate, bottom right shows the correlation between indoor and outdoor PM sulfate, and the top-right shows the degree of correlation (*R*^2^) for a lagged correlation between indoor and outdoor PM sulfate observations.

Figure 6 shows a time series of PM_1_ sulfate outdoors and indoors as measured by the JHU Aerosol Mass Spectrometer (AMS).^59,91^ A lagged correlation of these data (top right panel) shows that the best correlation is achieved with a delay of 40 min, which is an estimate of the typical transport time, indicating that the turnover time of air in the house under experimental conditions is on the order of an hour, which is faster than the forced leakage rate indicated. Future analysis will use other chemical tracers to better understand air exchange at the house and its dependence on environmental conditions. The bottom right subplot shows that the indoor sulfate is about 18% of the outdoor value. These findings show that infiltration filters most of the sulfate-containing particles out of the air, partially protecting the residents from this pollution. Future work will consider how different PM components and gases respond to infiltration.

### Indoor Sources and the Health-Related Properties of Indoor and Outdoor Air

A major sub-question of the above community question “How is the air quality in my house?” asks about indoor sources of pollution. Recent studies have investigated how cooking or cleaning activities affect indoor air quality.^30,31^ Because the focus of ALPACA is high latitude (Arctic) air pollution, we decided to focus on indoor sources related to heating, specifically use of a pellet stove.

When carrying out the pellet stove operations, we smelled smoke in the house, and instruments recorded high levels of smoke components (*e.g.*, organic PM, formaldehyde, furfural). We called the installer who came to the house and upon sliding the pellet stove insert out of the fireplace, we found a leaky gasket that was intended to seal the exhaust of the stove to the exhaust pipe through the wall. An attempted repair reduced the leakage into the house but did not completely solve the problem. This finding points out that it is important to maintain solid fuel combustion equipment and chimneys/exhausts to reduce indoor air pollution. Future studies will investigate if this problem was an uncommon one, or if similar issues exist for other installations.

Epidemiological studies indicate that adverse health outcomes are correlated with exposure to higher mass concentrations of fine particulate matter (PM_2.5_).^103,104^ However, the chemical composition and reactivity of these particles are also likely to affect their toxicity. Therefore, a number of assays aimed at improving linkages to adverse health effects have recently been developed,^105−107^ which may point to different pollution control strategies than those based on PM_2.5_ mass concentration.^108^ Reactive oxygen species (ROS) play a central role in chemical transformation of PM and adverse aerosol health effects upon inhalation and respiratory deposition of PM.^109^ Environmentally persistent free radicals (EPFRs) contained in atmospheric PM are shown to induce oxidative stress in living cells.^110^ EPFRs are often formed by incomplete combustion.^111^ Oxidative potential (OP) of PM represents the redox activity and is often used as an indicator of toxicity of the particles. It is commonly measured by the dithiothreitol (DTT) assay, which applies the underlying assumption that the DTT consumption rate would correspond to the rate of ROS formation.^112^

The UCI group collected PM samples in indoor and outdoor environments at the house site. Outdoor PM_2.5_ filter samples (prebaked 8 in. × 10 in. microquartz filters) were collected daily for 23.5 h using a Tisch Hi-Vol PM_2.5_ sampler. PM samples were collected indoors using a MOUDI cascade impactor (0.056–18 μm) during certain activities including pellet stove burning and cooking conducted in the house. The filter samples were analyzed for EPFRs, ROS, and OP using the DTT assay (OP^DTT^). We quantified EPFR and ROS molar concentrations and DTT decay rate per sampled volume of air to represent ambient concentration, which is related to level of exposure and calculated concentration per sampled PM mass, which represents a better metric for intrinsic PM health-related properties.

As a baseline for indoor experiments and to understand health-relevant measures of outdoor air, we measured outdoor concentrations of EPFRs per volume of air (EPFR_v_) outside the house to be 18 (± 12) pmol m^–3^, which is 1.3 times higher than previous measurements at an urban site in Irvine, CA,^86^ but 2 times lower than near highway sites in California,^86^ and lower than highly urban polluted cities in China, such as Linfen and Beijing.^113,114^ The average EPFR concentration per particle mass (EPFR_m_) was 1.4 (± 0.4) pmol μg^–1^, which is higher than ambient EPFR_m_ in Mainz,^115^ and highway and urban samples for PM_1_.^87^ The measured ROS concentration (ROS_V_) sampled in outdoor PM was 1.1 ± 1.0 pmol m^–3^, which is ∼9 times lower than those reported near highway sites in California^86^ but is comparable to ROS_V_ measured in wildfire PM in Irvine, CA.^87^ The average OP^DTT^ per sampled outdoor air was 254 (± 103) pmol min^–1^ m^–3^, which is lower than previous measurements in studies conducted in Irvine,^86^ but similar to measurements in Atlanta^116^ and the central California basin.^117^

Georgia Tech focused on measurements of OP^DTT^ for both the outdoor and indoor environments to assess health-relevant PM properties and how indoor air was affected by infiltration of outdoor air and by indoor sources. Table 4 shows the ratios of indoor/outdoor OPP^DTT^ either on a volume or mass normalized basis for background conditions and during indoor activities. Background conditions were defined as indoor air measurements at the start of the study, prior to any indoor activities, during which times the in/out OP_v_^DTT^ was on average 7%, which is significantly lower than the 18% infiltration found for sulfate, suggesting loss of species that contribute to OP by both transmission and temperature-driven volatility as the aerosol moved from outside to inside the house. The mean of the mass-normalized indoor and outdoor ratio, in/out OP_m_^DTT^, was about 50%, meaning that the DTT-based health-related properties of the infiltrated outdoor PM_2.5_ was reduced by about one half, although there was substantial variability in this ratio likely related to variability of outdoor particle composition and the infiltration process. Thus, the particle chemical composition that drives the OP^DTT^ was substantially changed when outdoor air infiltrated the house, and the process reduced indoor exposure (OP_v_^DTT^) levels to 7% of outdoor levels. Future analysis using the SV-TAG data will study evaporation of semivolatile species from the PM to understand what species may be responsible for loss of OP upon PM infiltration and the fate of the gases produced.

**Table 4:** Ratio of Indoors to Outdoors Oxidative Potential (OP) Determined by the DTT Assay by Georgia Tech Based on 24 h Filter Samples^a^

	*N*	In/Out OP_v_^DTT^	In/Out OP_m_^DTT^
Background	6	0.07 ± 0.05	0.53 ± 0.37
Pellet Stove	6	0.70 ± 0.38	1.57 ± 0.43
Incense	1	0.22	1.01
Cooking	2	0.15 ± 0.01	0.46 ± 0.16

a OP^DTT^ ratios are shown per volume of air (with v subscript) and per PM_2.5_ mass (m subscript). Background refers to samples taken at the start of the study prior to any indoor perturbations by various activities, which are also listed. *N* is the number of filter samples. Means and ± standard deviations are shown.

During indoor perturbation experiments involving various activities, indoor emissions from a pellet stove increased exposure levels beyond what would be expected by only infiltration of outdoor air (in/out OP_v_^DTT^ = 70%, which is greater than 7% for the background case). Pellet stove emissions also increased the proportion of adverse PM health-related components (proxy for toxicity) of the indoor particles relative to outdoors, shown in Table 4 by in/out OP_m_^DTT^ being greater than 1. In contrast, particles generated from cooking had much lower levels of the most health-relevant species, generally less than outdoor air, since in/out OP_m_^DTT^ < 1. These indoor experiments produced large increases in PM_2.5_ mass concentration, dominating the infiltrated particles; therefore, these in/out DTT results indicate that both the chemical composition and the amount of particles should be considered as a health metric for indoor air quality.

### Volatile Organic Compounds in Fairbanks Compared to Other Regions

Fairbanks residents know that PM_2.5_ air quality standards are violated during winter, but they often note different smells around the city and ask “What else is in the air?”. To this end, the group from LCE (Aix-Marseille Université), France, deployed a proton-transfer-reaction time-of-flight mass spectrometer (PTR-ToF-MS) downtown at CTC and sampled outdoor air for volatile organic compounds (VOCs). PTR-ToF-MS simultaneously detects of a wide range of trace VOCs (at pmol mol^–1^ level) with fast time resolution of seconds to minutes.^62^

Table 5 compares median VOC concentrations in downtown Fairbanks to two recent field studies in February and March 2018 in Boulder, CO and New York City, respectively.^118^ The results show that the distribution of VOCs differs greatly between downtown Fairbanks and these mid-latitude cities. The gasoline-related aromatic compounds such as benzene, toluene, and C8 aromatics are 4 to 12 times more concentrated in downtown Fairbanks than in Boulder or New York City. Additional VOC measurements performed during the ALPACA field study at the suburban UAF-Farm site (see section below) showed median concentrations of 0.15 and 0.23 nmol mol^–1^ for benzene and toluene respectively, hence substantially less than in downtown Fairbanks but still in the range of the New York study, which is remarkable since the UAF-Farm site is in an area of low population density. Cold start and idling emissions at low temperature likely contribute to the higher levels observed for these compounds. The lower median ethanol mixing ratio in downtown Fairbanks compared to the other studied cities is consistent with the use of ethanol-free gasoline because Alaska is exempt from the renewable fuel standard. VOC composition at Fairbanks is also more affected by compounds commonly associated with wood smoke.^119,120^ This difference is reflected by the level of acetonitrile (a common biomass burning tracer), which is roughly 5 and 15 times more concentrated than in New York and Boulder, respectively. The same effect with higher wood smoke components in downtown Fairbanks than other cities is observed for the main phenolic and furanic compounds released by wood combustion, with mixing ratios 6 to 12 times higher in Fairbanks for phenol and 5 to 25 for furfural. More comparable mixing ratios are observed for some oxygenated VOC such as acetone and methyl-ethyl-ketone (MEK), suggesting that these compounds come from other sources. Future work will further examine differences in VOC mixing ratios between downtown and the house site, outdoor and indoor sources of VOCs, their chemistry, and their partitioning between particle and gas phases.

**Table 5:** Median Mixing Ratios in nmol mol^–1^ of Selected VOCs in Downtown Fairbanks during ALPACA (LCE-France-CASPA) Compared to Recent Measurements in New York, NY and Boulder, CO^118^^a^

ionic formula	assignment*	New York, March 5–28, 2018	Fairbanks-CTC, Jan. 21-February 26, 2022	Boulder, February 1–18, 2018
C_2_H_3_NH^+^	Acetonitrile	0.049	0.197	0.0126
C_2_H_6_OH^+^	Ethanol	7.537	0.367	2.006
C_2_H_4_OH^+^	Acetaldehyde	0.633	1.464	0.259
C_3_H_6_OH^+^	Acetone	0.966	1.098	0.394
C_4_H_8_OH^+^	Butanone (MEK)	0.147	0.151	0.066
C_6_H_6_H^+^	Benzene	0.146	0.638	0.090
C_7_H_8_H^+^	Toluene	0.225	1.526	0.141
C_8_H_10_H^+^	C_8_ Aromatics	0.172	1.280	0.108
C_6_H_6_OH^+^	Phenol	0.011	0.068	0.006
C_5_H_4_O_2_H^+^	Furfural	0.012	0.057	0.002

a Assignment* represents the likely major VOC contributing to the signal at the corresponding ionic formula. This study (downtown Fairbanks CTC site) is in the middle column to facilitate easy comparison to neighboring columns.

### Chemical Transformations of Sulfur

Sulfate is a significant fraction of PM_2.5_ in Fairbanks winter, accounting for roughly 20% of the PM_2.5_ mass. This particulate sulfate comes from combustion of sulfur-containing fuels (*e.g.*, heating oil, coal, and aviation fuels). Therefore, there is a critical need to trace fuel sulfur to particulate sulfate so that appropriate regulatory approaches can be suggested to the community.

**Figure 7:**
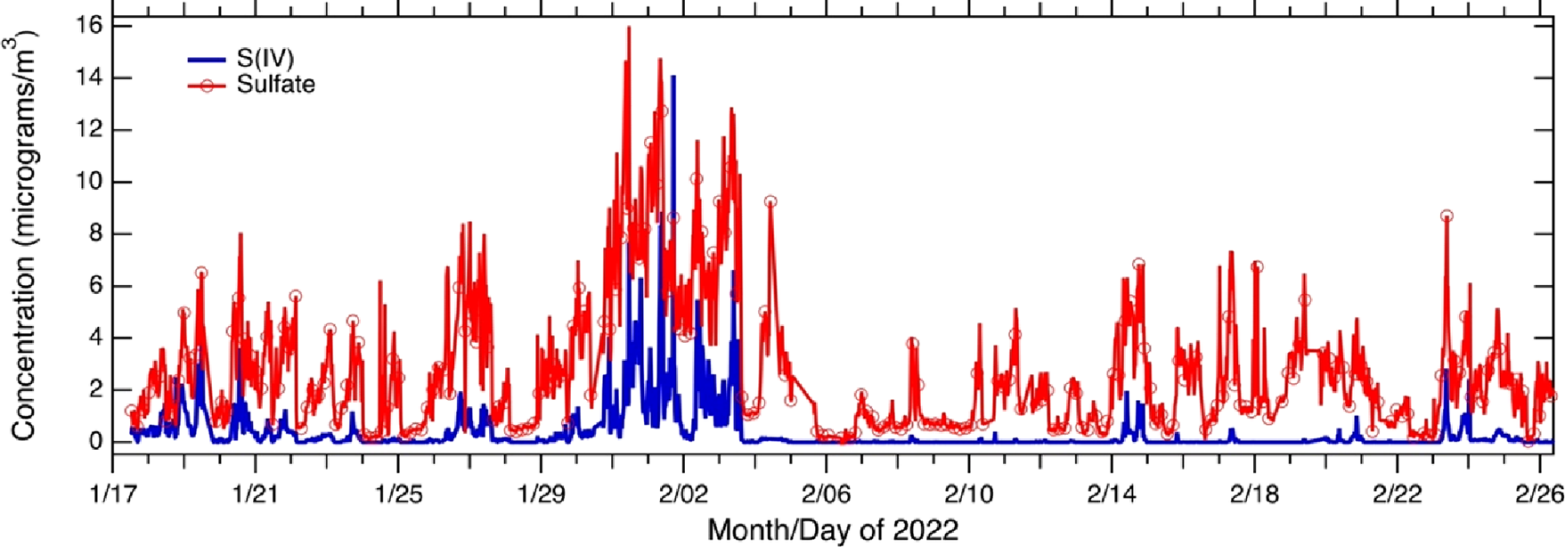
PILS-IC measurements of PM_2.5_ sulfate and S(IV) measured at the CTC site. The IC separation resolves sulfate from S(IV) species (sulfite, bisulfite, and organo-sulfite adducts such as hydroxymethanesulfonate, HMS), which co-elute. HMS is a component of the S(IV) species. Note that the ratio of S(IV) to sulfate varies in time, being high in the cold event (late January/early February), but low in the warm event in late February.

During poor air quality events, such as the “cold pollution event” highlighted in Figure 4, sulfate, shown on Figure 7, on average was 26% of PM_2.5_ mass but exceeded 40% for individual spikes. Analysis of sulfate isotopes in Fairbanks during the ALPACA field study showed that 62% ± 12% of sulfate came from primary sources, with a smaller influence of secondary chemistry.^121^ Besides increasing PM mass concentration, sulfate also affects the uptake of ammonia into particles, which indirectly further increases PM mass^122^ and can affect pH.^123^ In the past, sulfate was the only sulfur-containing species in PM that was quantified and reported by ADEC; however, it was noted that PM total sulfur by X-ray fluorescence analysis was on average about 10% larger than sulfur in the sulfate detected by ion chromatography, indicating a missing sulfur species.^48^ A study in the two winters prior to the ALPACA field study showed that S(IV) species, which include hydroxymethanesulfonate (HMS), were present in Fairbanks PM,^50^ potentially explaining this missing sulfur species. PM S(IV) species, in addition to sulfate, were measured during the ALPACA field study by particle into liquid sampling–ion chromatography (PILS-IC). PILS-IC can separate sulfate from the S(IV) species (organo-sulfite species such as HMS, sulfite, and bisulfite), but these S(IV) species were found to co-elute, preventing separate measurement of S(IV) species. Figure 7 shows PILS-IC measurements of PM_2.5_ sulfate and S(IV) throughout the study period, demonstrating large temporal variability for both species and that the S(IV) to sulfate ratio varies in time. During the “cold pollution event”, which had the campaign’s highest PM_2.5_ concentration (see Figure 4), both sulfate and S(IV) were high, and S(IV) reached its highest levels relative to sulfate for the complete study. During the “warm pollution event” at the end of the study, the ratio of S(IV) to sulfate is markedly lower. These results show that understanding not only sulfate, but also S(IV) and HMS formation, its chemical characteristics, and how it is linked to sulfate is needed for developing strategies to lower PM_2.5_ and to predict how regulations may affect future concentrations.

### Vertical Distributions of Pollution and Temperature over Downtown

Temperature inversions, where warm air lies above a pool of colder air, are common in Fairbanks, Alaska, during wintertime, and residents know that when there is an inversion it traps pollution and air quality gets worse downtown at the valley floor. However, the height to which pollution mixes was not known, and residents often asked about how high the pollution gets, and if the inversion affects downmixing of the plumes from power plants. To address this question, UCLA deployed a long-path differential optical absorption spectrometer (LP-DOAS), which uses light to remotely sense vertical distributions of gases from the 12–191 m above the valley floor.^73,77,124^

**Figure 8:**
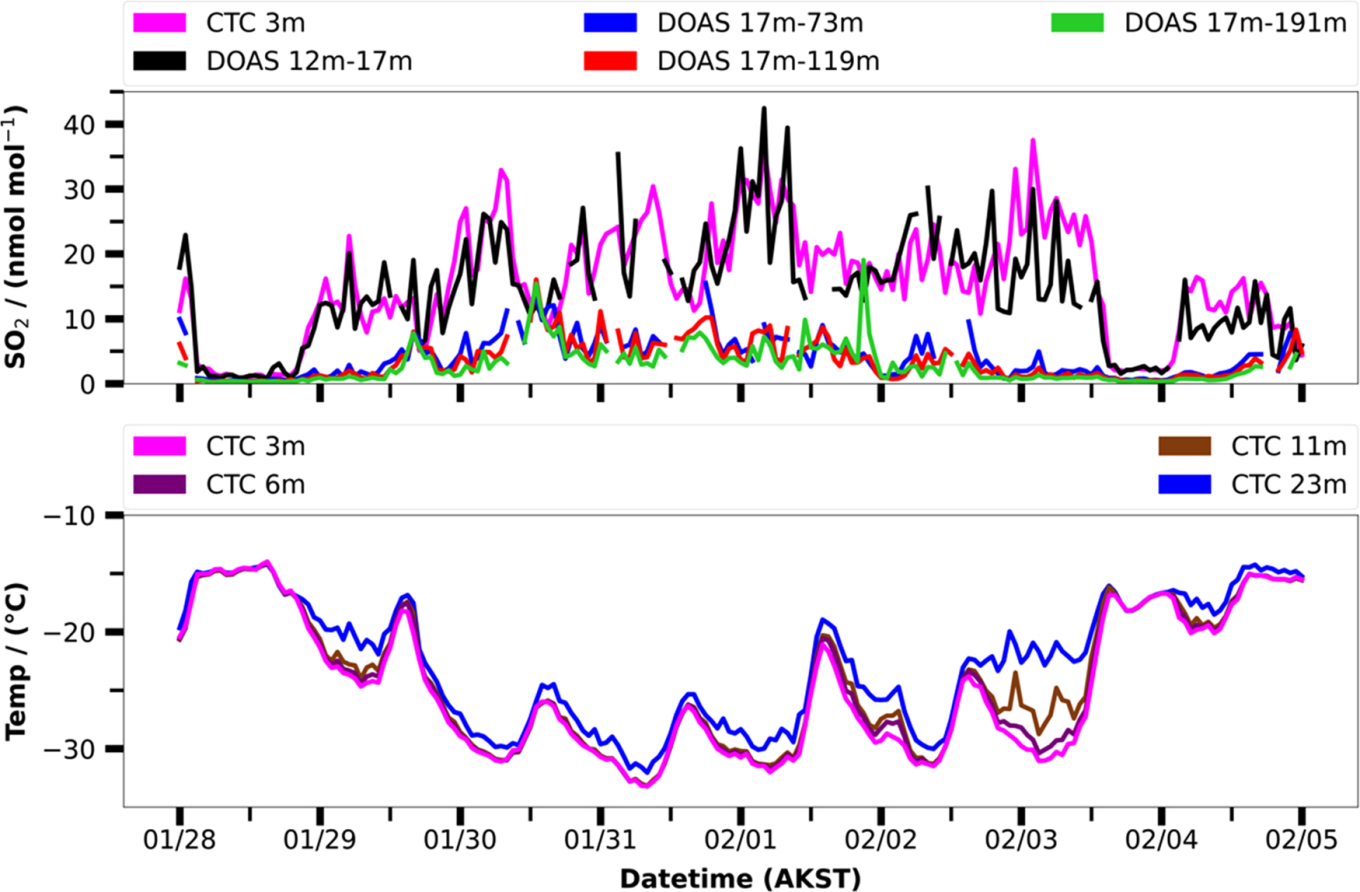
Vertical profiling of SO_2_ gas (averaged over the specified altitude interval) by the UCLA LP-DOAS, in-situ SO_2_ measurement at 3 m AGL and temperature measured between 3 and 23 m during the cold polluted event. The strength of the surface-based inversion can be visualized at the difference in temperature between 23 m and 3 m, which peaks at night, particularly during 1–3 January 2022.

Figure 8 shows path-averaged SO_2_ mixing ratios during the cold polluted episode in the top panel, along with the in-situ SO_2_ mixing ratio measured at CTC. The figure demonstrates that the lowest path, between 12 and 17 m above the valley floor, agrees well with the 3 m in-situ measurement, showing that there is a mechanically mixed polluted layer near the ground. Figure 8 also shows the temperature stratification measured by aspirated thermometers^36^ at CTC. Temperature differences between building top to ground were used as an indicator of the SBI strength. Figure 8 shows that enhanced SO_2_ mixing ratios occur when there is a temperature inversion and also become larger as it gets colder in this episode. However, the paths viewing upwards to reflectors on Birch Hill show much smaller path-averaged concentrations, indicating that there is less SO_2_ aloft than in the polluted surface layer. The observations show that for most of the strongly stable periods, the path average from 17 to 73 m above the valley floor is a small fraction of the surface mixing ratio, demonstrating that the pollution trapping height is well below 73 m. If this pollution were coming from aloft, then larger path-averaged mixing ratios on upper paths would be expected, which are not generally observed for SO_2_ near downtown.

This work indicates that in downtown Fairbanks, most pollution at breathing level comes from ground-level sources. However, in other places around the Fairbanks North Star Borough or at other times, there may be downwash of power plant plumes. We are working on modeling these situations with the aim to have a picture of the relative contributions of elevated point sources versus ground-level sources to pollution at the surface.

### Intercepts of Lofted Pollution by Tethered Balloon Sampling

Power plants in Fairbanks are major emitters of SO_2_, NO_*x*_, PM, CO, and CO_2_, so the potential impact of these emissions on ground level air quality, their downwind processing, and eventual air pollutant deposition is an important issue. At times when the downtown power plant plumes are blowing to the west, the plumes could descend as they cross to West Fairbanks. Therefore, residents often ask how often downwash happens, where it happens, and how much influence it has on breathing-level air quality. To address this, the Swiss (EPFL) group, together with French (CNRS) and Italian (CNR) groups, flew the EPFL profiling tethered balloon system (Helikite)^74^ with various particle and gas sampling payloads from the UAF-Farm site in West Fairbanks.

**Figure 9:**
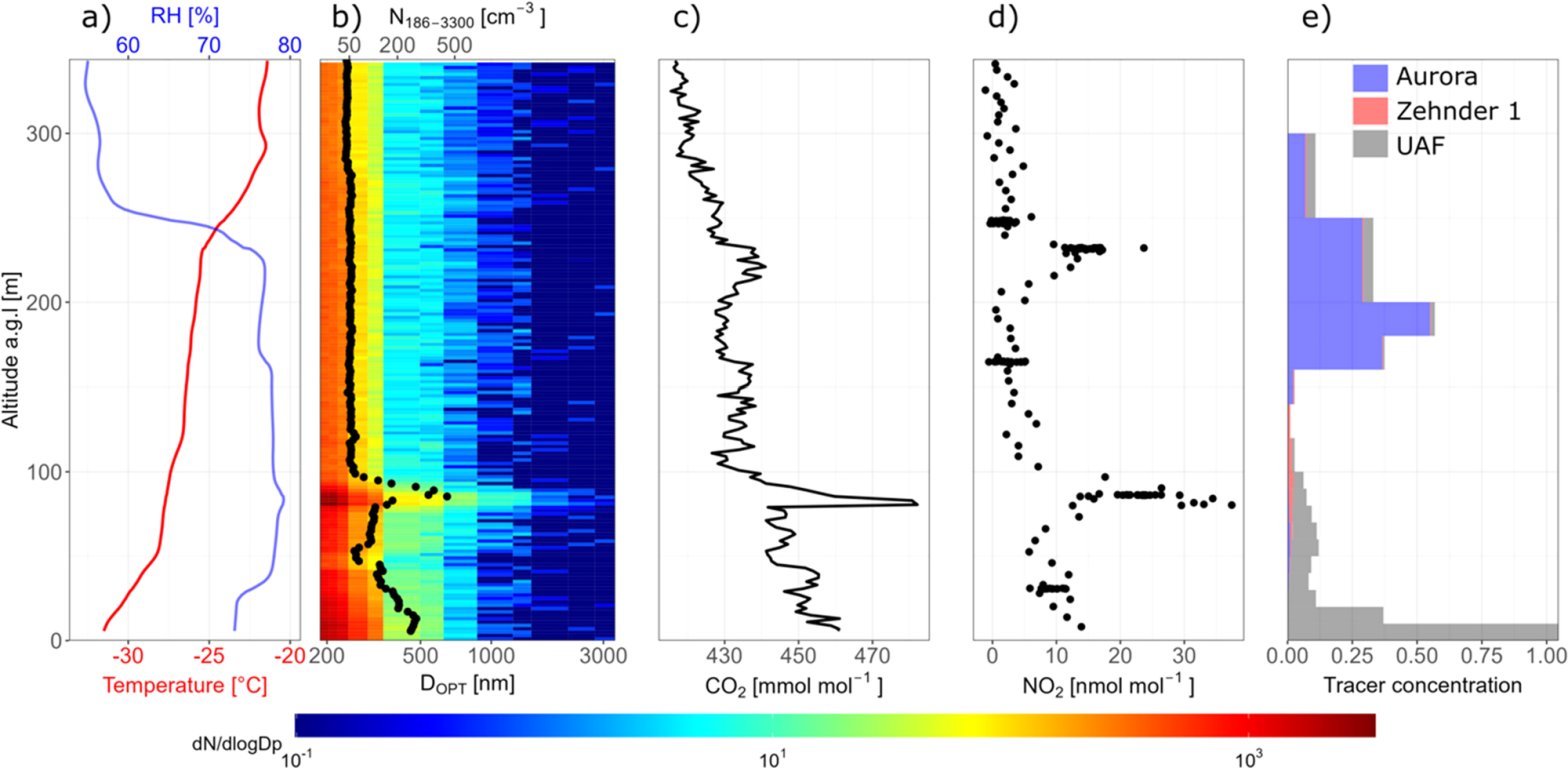
Helikite profile of the vertical structure of the atmosphere from 09:50 to 10:35 AKST on January 30, 2022. (a–d) Temperature, RH, particle counts, and CO_2_ are part of the Helikite measurement payload, and NO_2_ was measured with the LAERO-CNRS (CASPA) MicroMegas package. The right-most panel (e) shows power plant tracer forecasts for January 30, 2022 as a function of altitude at the UAF Farm site. On this day, tracers originating from the UAF and Aurora power plants, along with a small contribution from Zehnder power plant were forecast to arrive over the UAF Farm site. See text for details.

Figure 9 shows an example vertical profile measured on the Helikite platform from 09:50 to 10:35 AKST on January 30, 2022. The payload captured profiles of temperature, relative humidity (RH), particle number concentrations between 0.186-3.3 μm, CO_2_, and NO_2_. The right-most panel (e) shows power plant tracer forecasts at 10 AM on this day above the UAF-Farm site produced using a Lagrangian dispersion model (see below). Six powerplants in the region were included in the simulation, but only three were predicted to influence this site.

This profile shows a surface-based inversion (SBI) reaching an inflection at around 50 m, and at this point the particle number concentration reaches a local minimum. The 0.186–3.3 μm particle number concentrations range from about 220 cm^–3^ at the ground to 50 cm^–3^ at 50 m. Similarly, CO_2_ and NO_2_ decline with increasing altitude in the surface-based inversion layer. Above 50 m, the atmosphere continues to be stable but is less stable than the SBI. This near isothermal layer is capped with a more stable elevated temperature inversion layer from 250–300 m. During this profile, the Helikite crossed a thin pollution plume between 75 and 100 m. The plume is characterized by high particle number concentrations and increased CO_2_ and NO_2_ mixing ratios. The peak concentration enhancements within the plume are significantly higher than concentrations within the surface-based inversion layer at the UAF-farm site, indicating that if the plume descended to the surface, it could increase surface mixing ratios. Further aloft, between 200 and 250 m, we recognize another plume, albeit with weaker pollutant enhancements, characterized by increased CO_2_ and NO_2_ mixing ratios, but no enhancement in particles.

During the campaign, the FLEXPART-WRF Lagrangian particle model^80^ was run in forward mode with power plant emissions (provided by EPA/ADEC) for selected sources. Weather Research Forecast (WRF) forecasts from EPA were used to drive the simulations. The results were examined during the campaign and used to help with planning the Helikite flights and for initial data analysis. Results for the January 30 profile shown in Figure 9 indicate that several plumes were forecast to be over UAF-Farm site. A plume primarily from the UAF power plant is forecast between 50 and 100 m, and a plume from the downtown Aurora plant is forecast between 150 and 250 m. In this case, the forecast altitudes appear similar to the intercepted plumes. Surface emissions (*e.g.*, home heating, transportation, etc.) were not included in this model configuration; therefore, it is not possible to assess the relative contribution of lofted and ground-based sources to breathing level air quality in these early simulations. Future work, both using FLEXPART-WRF and an Arctic version of WRF-Chem,^79^ combined with a plume rise parameterization for each power plant and incorporation of high resolution surface emissions, will model the extent to which lofted sources affect ground-level pollution.

### Relationship between Downtown and UAF-Farm Ground-Level Pollution

**Figure 10:**
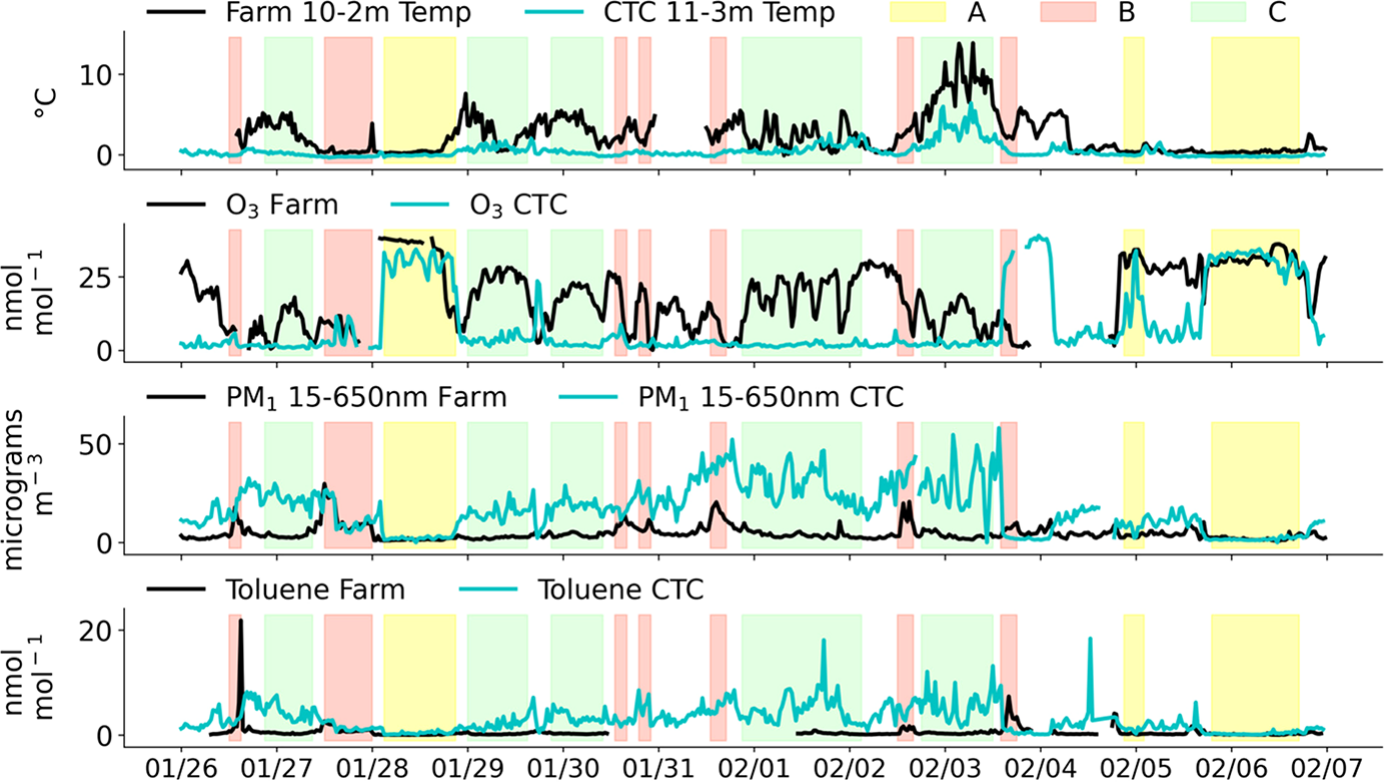
Comparison of near-surface temperature difference over 8 m vertical difference (SBI strength), ozone, PM, and toluene at the UAF-Farm site and the downtown CTC site. Periods labeled “A” are cleaner periods, “B” are periods of outflow from downtown to the UAF-Farm, and “C” periods are more stagnant and have greater pollution downtown. Toluene was measured using LCE (France) CHARON PTR-ToF-MS at CTC and CNR (Italy) miniCG Pyxis BTEX at the UAF-Farm.

It is well understood in Fairbanks that pollution levels vary spatially around the Fairbanks bowl. Robinson and colleagues^94^ used mobile monitoring in East Fairbanks to characterize the distribution of pollution and found that during strong inversion conditions hotspots were more intense and smaller, and the gradient in pollution between the valley and hills was stronger. The background areas on the hills north of Fairbanks experienced higher particle number concentrations on the days of weak inversions because of enhanced transport from hotspot areas in the city. Such findings compare well with the ground-based observations performed in West Fairbanks at the UAF-Farm location. Figure 10 shows a comparison of ground-based pollution measurements at the UAF-Farm site and at the downtown CTC site. UAF-Farm generally has much shallower SBIs than CTC. This may be due to the influence of cold flows from a nearby valley^39,40^ and the fact that the height of obstructions at the UAF-Farm is much lower than downtown. The UAF-Farm is a flat field for hundreds of meters, while downtown has an “urban canopy” consisting of buildings and trees, which are roughly 10–15 m tall. Eddies around those trees and buildings probably act to vertically mix downtown, reducing the inversion strength. There might also be an urban heat island where building heat leakage warms the near surface air,^125^ reducing temperature inversion strength.

Ozone in Fairbanks comes from background air from around the urbanized area. Pollution-sourced nitric oxide (NO) reacts with ozone, which removes ozone from polluted air masses, typically leading to near zero ozone during polluted periods. Surface ozone at the UAF-Farm nearly always exceeds ozone in downtown, which would be consistent with greater surface pollution at CTC compared to the UAF-Farm. Late on February 3, CTC cleaned out in a sharp transition where warm air that had been moving over the CTC (downtown) site eventually eroded the inversion and mixed to the ground, bringing ozone, and much warmer air. It appears that the inversion did not vertically break at the UAF-Farm until about half a day later, and maybe the clean air never downmixed there. Examining PM in the 15–650 nm size range, we see that PM concentrations are often lower at the UAF-Farm with respect to CTC, only peaking episodically (in the periods labeled “B” in Figure 10). At those times, PM rises to the CTC value, which would be consistent with UAF-Farm being downwind of CTC, or in an “outflow” region from polluted downtown. Measurements of toluene generally agree with PM, with elevated amounts on January 26 and February 3 providing evidence of transport of air from downtown to the UAF-Farm site. These aspects are being investigated further to understand the spatial distribution of pollution around the Fairbanks area.

## Conclusions

Wintertime pollution in urbanized areas is a serious problem for residents living in these areas.^1^ The ALPACA project started by documenting open questions asked by the community and knowledge gaps in the scientific literature that hindered answering these questions. This manuscript documents our multipronged approach, which involved work in three focal areas: (1) Investigations into outdoor air chemical processes at a downtown supersite located at the UAF-CTC building. (2) Measurements of particle and gas vertical profiles to understand their relationship to meteorological processes and their dispersion rates. (3) Studies of air outside and inside a residential house to understand infiltration, changes to particles and gases upon warming associated with migration indoors, and indoor sources of particles and gases, which interact with the infiltrated air. The indoor studies involved both passive observations of indoor and outdoor air without experimental manipulation of the house and intentional experiments seeking to understand indoor sources (e.g., heating with the pellet stove and cooking) and their interactions with indoor air components.

The field campaign was carried out during January and February 2022, which was somewhat abnormal because of the peak COVID case rate and a very large rain-on-snow event which made travel difficult. However, analysis of the distribution of hourly PM_2.5_ mass concentration measurements in this period places it well within the variability of recent winters pollution levels. A variety of conditions were encountered, but in particular, the campaign sampled a cold polluted event that had ice fog and exceeded 24-h EPA pollution standards, and a contrasting warm polluted event, which despite temperatures being close to freezing, had high sustained PM loading.

The study was motivated by community questions, so in this manuscript, we highlighted initial results of the study related to those questions. We found that air that had infiltrated into a test house had significantly reduced PM sulfate, indicating loss of particulate matter upon infiltration through the building envelope. We found that the indoor sources of heating with the pellet stove and cooking can lead to higher indoor PM loadings than outdoors, and that the particles from the pellet stove had a larger oxidative potential (a proxy for adverse health effects) than outdoor particles and cooking particles. Outdoor particles were found to have higher environmentally persistent free radical (EPFR, another proxy for adverse health effects) concentrations than California and German cities, but less than a roadside location in California and Chinese cities. Given that indoor activities are highly variable and we only studied one house, it is not possible to generalize these indoor air quality results to all houses in cold climates, but they point out that indoor sources are likely to dominate infiltration of outdoor air as sources of PM and oxidative potential.

Measurements of VOCs allowed us to relate Fairbanks winter air composition to other cities and showed that many gases are significantly more concentrated in Fairbanks than lower-latitude cities. These increased mixing ratios are probably a combination of strong sources and trapping of pollution related to the cold climate, and possibly reduced sinks by slower chemistry. During periods of strong temperature inversions, we found that path-averaged SO_2_ between 12 and 17 m above the valley floor agreed well with 3 m inlet in-situ measurements, but that SO_2_ on the next higher path, which was between 17 and 73 m, was greatly reduced. This indicates that the pollution is trapped on scales significantly below 73 m when there is a strong temperature inversion. Tethered balloon measurements intercepted pollution plumes aloft that are attributed to power plant emissions, which had high pollution concentrations and generally limited vertical extent. Various plumes had different ratios of particles and gases. Power plant plume tracer forecasts indicate some influence of power plant plumes on surface air quality, but further quantification is needed. Future work will use modeling to determine how often downwash happens and constrain its influence on breathing air quality, as well as examining recirculation of surface and power plant pollution. Measurements at different horizontal locations around the Fairbanks bowl as well as mobile studies^94^ give insight into source and receptor locations and the combination of horizontal and vertical dispersion of pollution around the Fairbanks area.

The ALPACA science team is currently working on manuscripts that delve deeper into the observational data captured in this study. Those studies promise to improve understanding of the unresolved questions described above. Other aspects of the greater ALPACA project involve modeling of these results and performing laboratory studies that seek to improve the representation of chemical and physical processes in models. The ALPACA project also had a social science component, which surveyed the community to assess public preferences for how to address these problems and attitudes toward and knowledge about the air quality problem. Those social science studies are critical to assisting the community in crafting solutions to these problems. To that end, the whole project is embedded in community connection activities that will help the community, regulators, and other stakeholders to improve wintertime air quality.

ALPACA represents the first, large-scale international experiment characterizing air pollution in a high-latitude (near Arctic) city. The results highlight the strong connections between winter climate conditions, energy production and use, meteorological effects on pollutant dispersion and sinks, atmospheric chemistry, and pollutant transformations and can inform further assessments of the nature of atmospheric pollution at other Arctic urban locations. The warming Arctic may lead to increased human activity, for example, increased resource extraction, and urbanization making it important to understand these issues.

## Data Availability

Final data from the study will be available to the scientific community through the ALPACA data portal hosted by Arcticdata.io (https://arcticdata.io/catalog/portals/ALPACA).
